# Genomic structure, expression pattern, and functional characterization of transcription factor E2F-2 from black tiger shrimp (*Penaeus monodon*)

**DOI:** 10.1371/journal.pone.0177420

**Published:** 2017-05-30

**Authors:** Bobo Xie, Pengfei Wang, Chao Zhao, Lihua Qiu

**Affiliations:** 1 South China Sea Fisheries Research Institute, Chinese Academy of Fishery Sciences, Guangzhou, PR China; 2 College of Aqua-life Science and Technology, Shanghai Ocean University, Shanghai, PR China; 3 Key Laboratory of South China Sea Fishery Resources Exploitation & Utilization, Ministry of Agriculture, Guangzhou, PR China; 4 Tropical Aquaculture Research and Development Center of South China Sea Fisheries Research Institute, Sanya, PR China; Shanghai Ocean University, CHINA

## Abstract

Transcription factor E2F-2 is a regulator of cell cycle. Researchers identified *E2F-2* genes from yeasts to humans, but few reports investigated *E2F-2* gene from black tiger shrimp. In the present study, we cloned *E2F-2* gene from black tiger shrimp (*Penaeus monodon*). Full-length *PmE2F-2* complementary DNA sequence measures 3,189 bp with an open reading frame of 1,371 bp. Complete *PmE2F-2* genomic sequence (17,305 bp) of *P*. *monodon* contains nine exons, which are separated by eight introns. Quantitative real-time polymerase chain reaction (qRT-PCR) analysis indicated that *PmE2F-2* is highly expressed in hepatopancreas and ovaries of *P*. *monodon*. Highest *PmE2F-2* expression levels were observed in stage III ovarian development of *P*. *monodon*. *PmE2F-2* expression levels were significantly augmented in ovaries of *P*. *monodon* after 5-hydroxytryptamine injection and eyestalk ablation. RNA interference experiments were conducted to examine *PmE2F-2*, *PmCDK2*, and *PmCyclin E* expression profiles. *PmE2F-2* was successfully knocked down in ovaries and hepatopancreas via double-stranded RNA (dsRNA)–E2F-2 injection. In the same organs, *PmE2F-2* expression localization and level were investigated through in situ hybridization, which revealed consistent results with those of qRT-PCR. After dsRNA—E2F-2 injection, gonadosomatic index of shrimp was significantly lower than those following dsRNA—GFP and phosphate-buffered solution injections. Therefore, *PmE2F-2* may be involved in ovarian maturation in *P*. *monodon*.

## Introduction

The E2F transcription factor family was first studied in 1980s as an adenovirus E2 gene promoter activator [1]. Currently, eight E2F family member genes are known, from E2F-1 through E2F-8 [2]. E2Fs significantly contribute in gene expression regulation during G1-/S-phase transition of the mammalian cell cycle [3,4]. Overexpression of E2F-1 or E2F-4 transforms rat embryo fibroblasts, the transcription factor E2F is required for S phase during Drosophila embryogenesis [5]. In both sexes, early gonadal development is characterized by the migration of extraembryonically derived primordial germ cells into the surface epithelium and underlying mesenchyme of the mesonephros and the appearance of the sexually indifferent gonad or genital ridge. Several genes are now known to have definitive roles in gonadal development and sex differentiation; they include steroidogenic factor1 (SF1), the testis-determining gene (SRY), Wilms’ tumor antigen (WT1), and Müllerianinhibiting substance (MIS) [6]. E2F family plays a crucial role in cell proliferation, differentiation, and apoptosis and is associated with pRb and other cell-cycle-dependent proteins [7]. Rb functions as cell cycle repressor by inhibiting activity of E2F transcription factor. Hyperphosphorylated Rb releases E2F and promotes expression of genes mediating entry into S phase [8,9]. Given that cyclin E gene is E2F responsive, cyclin E/cdk2 complexes act through positive feedback loop to facilitate progressive pRb phosphorylation and further E2F release, resulting in rapid rise in cyclin E/cdk2, which allow cells to initiate DNA replication [10]. E2F activities were described in vast majority of studied eukaryotes, ranging from plants to mammals, such as *Homo sapiens* [11], *Mus musculus*, and *Danio rerio* [12]. However, E2F is still undescribed in black tiger shrimp (*Penaeus monodon*), and during oocyte maturation, regulatory roles of E2F remain poorly understood.

RNA interference (RNAi) was applied to clarify gene functions in shrimp. For example, knockdown of *Pmp53* expression indicates that *Pmp53* may play an important role in ovarian development of *P*. *monodon* [13]. Gonad-inhibiting hormone transcripts are silenced to exploit efficient RNAi-based techniques, which stimulate gonadal development and spawning in *Litopenaeus vannamei* [14]. Knockdown of enolase demonstrates its importance in white spot syndrome virus infection in kuruma shrimp [15]. In the present study, RNAi was used to verify the function of *E2F-2* gene (*PmE2F-2*) in ovarian development of *P*. *monodon*.

*P*. *monodon* is a commercially important aquaculture species in South China and Southeast Asia. Eyestalk ablation can induce ovarian maturation in *P*. *monodon*, but this process can result in reduction in egg quality and death of spawners [16]. Therefore, studying cell cycle regulation can help further our understanding of molecular mechanisms underlying ovarian development and maturation of *P*. *monodon* [17]. To examine molecular mechanisms of *E2F-2* gene involvement in ovarian development of *P*. *monodon*, we successfully cloned *E2F-2* full-length complementary DNA (cDNA) and genome from *P*. *monodon*. We also investigated expression patterns of *PmE2F-2* transcripts in different tissues and ovarian developmental stages. We characterized relative expression profiles in response to double-stranded (ds) RNA—E2F-2 and 5-hydroxytryptamine (5-HT) injections and eyestalk ablation. Lastly, we revealed possible mechanism of *E2F-2* gene involvement in ovarian development of *P*. *monodon*.

## Materials and methods

### Experimental animals and sample preparation

Experimental shrimp (36 ± 3 g body weight) specimens were collected from cultured populations in the Shenzhen base of South China Sea Fisheries Research Institute (Guangdong, China) and acclimated in aerated seawater (salinity 30) for three days at 24–26°C. About two thirds of the water in each tank was renewed daily. We randomly collected 3 experimental shrimps from each tank. Tissues were collected from three healthy shrimp, snap frozen in liquid nitrogen, and stored at −80°C; tissue samples included those from the muscle, heart, hepatopancreas, ovary, gill, brain, stomach, and intestine.

### Total RNA extraction and first-strand cDNA synthesis

Total RNA of dissected tissues was extracted using TRIzol reagent (Invitrogen, USA) based on manufacturer’s protocol. Total RNA integrity was verified through 1.2% agarose gel electrophoresis, and RNA concentration was determined with NanoDrop-2000 (Thermo Fisher, USA). First-strand cDNA was synthesized from 1 μg of total RNA using PrimeScript Reverse Transcriptase Kit (TaKaRa, Dalian, China).

### cDNA full-length cloning through rapid amplification of cDNA end (RACE)

Partial *E2F-2* gene sequence was obtained from transcriptome database. 3′ RACE—polymerase chain reaction (RACE—PCR) was performed using the gene-specific primer E2F-2–3GSP 1/2 and Universal Primer Mix (Table 1). RACE—PCR products were purified using PCR purification kit (Sangon Biotech, China), ligated into pMD18-T vector (TaKaRa, Dalian, China), and sequenced (Invitrogen, Guangzhou, China).

**Table 1:** 

name	primer sequence (5’→3’)	application
E2F-2-3GSP1	TGCTGTGAAAGCGCCACCTGGAA	3’RACE
E2F-2-3GSP2	TGCTGAGTGCTGCCCCTCATCCTT	3’RACE
qE2F-2-F	AGATGATGACAGTATCCGTAGT	Real time RT-PCR
qE2F-2-R	GAATGGTGGTGGTGAAGAC	Real time RT-PCR
T7-1F	AGCTTGGATCCTAATACGACTCACTATAGGGAGAG	
T7-1R	TCGACTCTCCCTATAGTGAGTCGTATTAGGATCCA	construct pD7 vector
T7-2F	AATTCGGATCCTAATACGACTCACTATAGGGAGAGAGCT	
T7-2R	CTCTCCCTATAGTGAGTCGTATTAGGATCCG	
pF	CAGTGAGCGAGGAAGCGGAAG	pUC18
pR	GGATGTGCTGCAAGGCGATTAAGT	pUC18
iE2F-2-F	CGAGCTCCCACGAAGACTGCCAGACGTGCTA	RNAi
iE2F-2-R	ACGCGTCGACGACAGCTTGTCAGAGGCCCTATTGA	RNAi
iGFP-F	CGAGCTCTGGAGTGGTCCCAGTTCTTGTTGA	RNAi
iGFP-R	ACGCGTCGACGCCATTCTTTGGTTTGTCTCCCAT	RNAi
Full-F	ATGGATAGCGTTGGGACC	Genome full length
Full-R	AAATCAAAGAGATCACTT	Genome full length
GSP1	GGTAGCTCAGTCTTTATGTTA	Genome walking
GSP2	TAAGGAGCCCAAGGGAGGTGT	Genome walking

### Genomic DNA isolation

Genomic DNA was prepared from muscle tissues using a previously described standard method [18]. DNA concentration was determined with NanoDrop-2000 (Thermo Fisher, USA).

### Characterization of genomic structure and promoter region of *PmE2F-2*

*PmE2F-2* gene sequence was acquired using a PCR-based strategy with genomic DNA as template and full-F and full-R primers (Table 1). Expected DNA fragment was obtained through a previously described standard method [19].

To determine the 5′ upstream sequence, BD GenomeWalker Universal Kit (Clontech, USA) was utilized based on manufacturer’s protocol, and its specific methods were carried out based on a previously described standard technique [19]. Whole *PmE2F-2* gene fragment was amplified using PCR with gene-specific primer (GSP) 1 and GSP2 (Table 1).

Neural Network Promoter Prediction was used to predict putative promoter and transcription start site of *PmE2F-2* (http://www.fruitfly.org/seq_tools/promoter.html) [20]. Transcription factor binding sites were predicted using Transcription Element Search System (http://www.cbil.upenn.edu/tess) [21].

### 5-HT challenge and eyestalk ablation assay

Neurotransmitters like serotonin (5-HT) is involved in the regulation of ovarian maturation and ovulation. To examine effects of 5-HT on *PmE2F-2* mRNA expression, 5-HT creatinine sulfate (Sigma, MO, USA) was dissolved in the sterilized saline solution and made into a 0.25 μmol solution. The first abdominal segment of female shrimp was injected intramuscularly with 50 μL 0.25 μmol 5-HT. Other shrimps were injected with sterilized saline solution (10 mM Tris—HCl at pH 7.5, 400 mM NaCl) at 0 h and were used as control. Ovaries were collected at 0, 6, 12, 24, 48, 72, and 96 h postinjection, snap frozen in liquid nitrogen, and stored at −80°C.

After unilateral eyestalk ablation, ovaries of female shrimp were collected at 0, 3, 6, 12, 24, 48, 72, and 96 h, snap frozen in liquid nitrogen, and stored at −80°C to analyze effects of the process on PmE2F-2 mRNA expression.

### RNAi assay

The specific primers that contained the T7 promoter site for RNAi experiments were designed using Snap Dragon tools (http://www.flyrnai.org/cgi-bin/RNAi_find_primers.pl). We design the primers (iE2F-2-F/R and iGFP-F/R) for dsRNA synthesis. The Sac I cleavage site was added at the 5-terminus of iE2F-2 / iGFP-F, and the Sal I cleavage site was added at the 5-terminus of iE2F-2 / iGFP-R. Difference in silencing effect was not observed between dsRNAs produced through in vivo bacterial expression and in vitro transcription [13]. Recombinant plasmids (pD7-E2F-2 and pD7-GFP) were established. For in vitro transcription, sense and antisense DNA templates were generated via PCR using pD7-E2F-2 and pD7-GFP recombinant plasmids as templates. dsRNA—E2F-2 and dsRNA—GFP were synthesized in vitro with Transcription T7 Kit (TaKaRa) following manufacturer’s instruction. dsRNA was stored at −80°C.

We chose the stage II of developmental stages of ovarian in *P*. *monodon* as the experimental shrimps. *P*. *monodon* samples were acclimatized for two days before dsRNA—E2F-2, dsRNA—GFP, and phosphate-buffered solution (PBS) injections. RNAi experiments were performed based on a previously describe standard method [13]. At 0, 6, 24, 48, 72, and 96 h postinjection, ovaries and hepatopancreas were collected and weighed from shrimps injected with dsRNA—GFP, dsRNA—E2F-2, and PBS, snap frozen in liquid nitrogen, and stored at −80°C. Gonadosomatic index (GSI, ovarian weight / body weight × 100) of each shrimp was calculated.

### In situ hybridization

Specific digoxigenin-labeled RNA probes against *PmE2F-2* were synthesized by the TaKaRa Company (TaKaRa, Dalian, China). At 24 h postinjection of dsRNA—E2F-2 and dsRNA—GFP, in situ ovary and hepatopancreas hybridization experiments were performed based on a previously described standard method [22].

### Sequence analysis

Full-length *PmE2F-2* cDNA sequences were analyzed using the Basic Local Alignment Search Tool programs at the National Center for Biotechnology Information (http://blast.ncbi.nlm.nih.gov/Blast.cgi). Complete open reading frame (ORF) regions and amino acid (aa) sequences were analyzed with ORF Finder (https://www.ncbi.nlm.nih.gov/orffinder/). Protein domains were predicted using Simple Modular Architecture Research Tool (http://smart.embl-heidelberg.de/). Multiple sequence alignments were created using Clustal W software (http://www.clustal.org/). Phylogenetic tree was constructed through neighbor-joining method in MEGA 5.03.

### Quantitative real-time PCR (qRT-PCR)

qE2F-2-F/R primers (Table 1) were used during qRT-PCR to detect temporal expression of *P*. *monodon*. qRT-PCR was performed using SYBR Premix Ex Taq II (TaKaRa, Dalian, China), and its specific methods were carried out based on a previously described standard method [23].

### Statistical analysis

Relative mRNA expression levels were examined through one-way analysis of variance (PASW Statistics 18.0; Chicago, IL, USA) in SPSS 22.0. P < 0.05 was considered statistically significant.

## Results

### Cloning and characterization of full-length *E2F-2* gene cDNA

**Fig 1:**
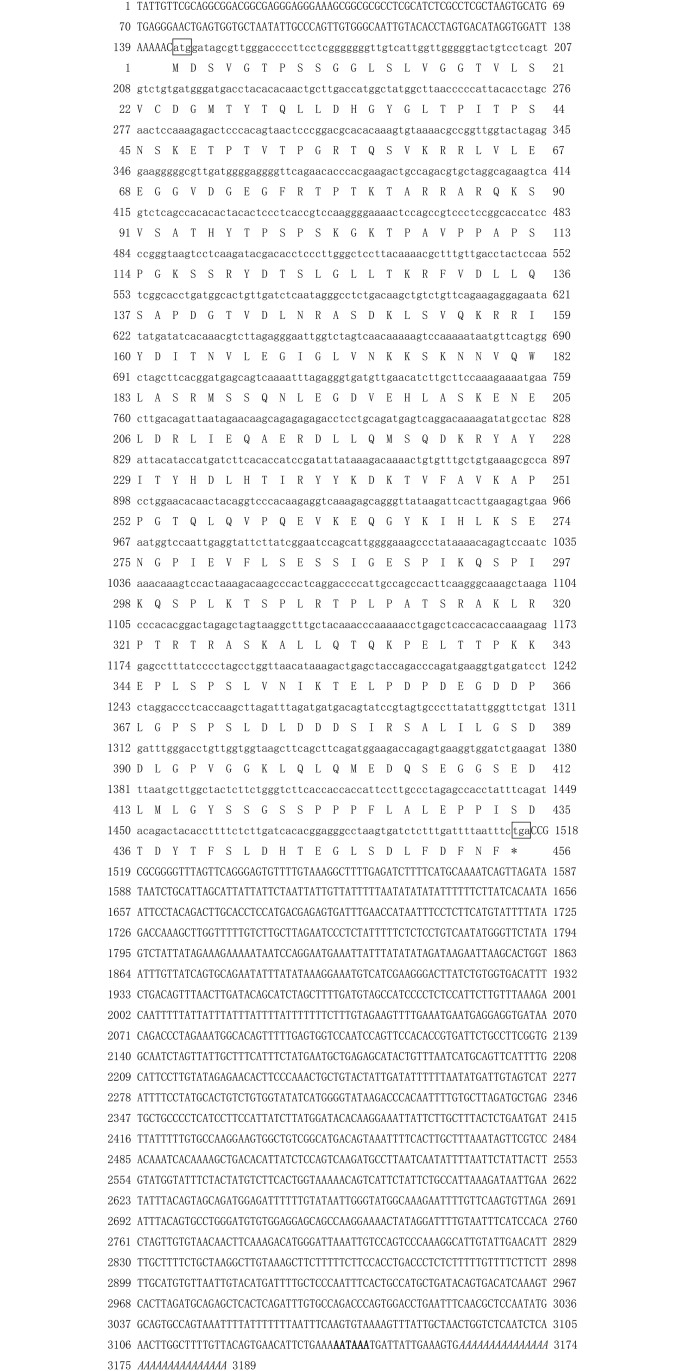
Nucleotide and deduced amino acid sequence of *PmE2F-2*. The deduced amino acid sequence is shown below the nucleotide sequence. The initiation code (ATG) and the termination code (TAA) are indicated by the box. The polyadenylation signal sequence (AATAAA) is in bold.

Full-length *E2F-2 gene* cDNA sequence (GenBank accession no.: KY628943) from *P*. *monodon* measures 3,189 bp and contains 144 bp 5′-untranslated region (UTR), 1674 bp 3′-UTR, and 1,371 bp ORF, which encodes for 456 aa with calculated molecular mass of 49.66 kDa. A 30 bp poly(A) tail is also found downstream of the gene. The deduced aa of *E2F-2* genes contain conserved E2F_TDP (119–184 aa), Rb_C (86–203 aa), and coiled-coil domain (189–225 aa) (Fig 1).

### Phylogenetic analysis of E2F-2 genes

**Fig 2:**
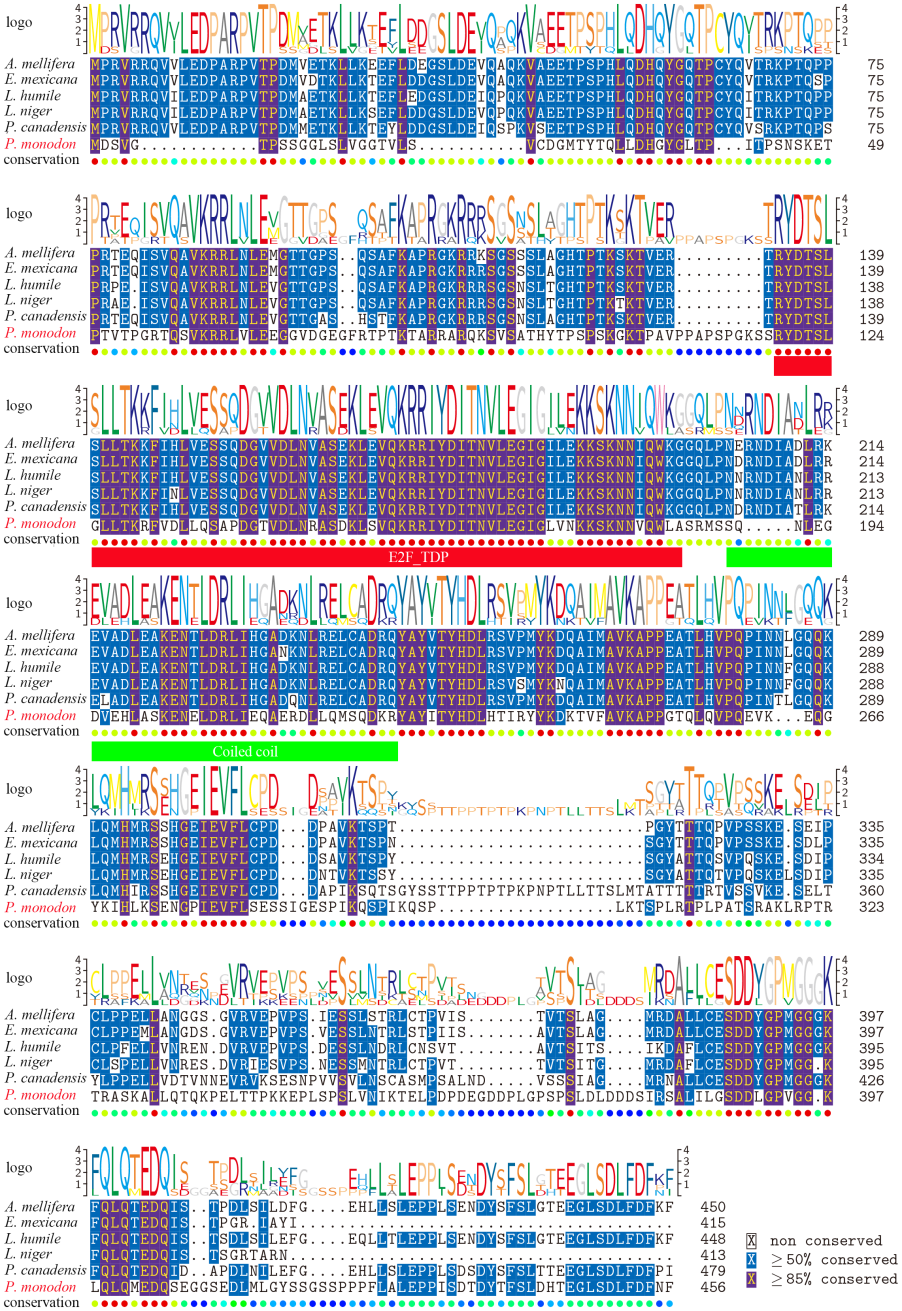
Multiple alignment of the deduced amino acid sequences of E2F-2 from *P*. *monodon* and other species. Sequence logo representing the similarity is shown at the top of alignments and numbers of amino acid are listed on the right side of alignments. The GenBank numbers of E2F-2 are listed as follows: *P*. *monodon*: KY628943; *Apis mellifera*: XP_006561713.1; *Eufriesea mexicana*: OAD58031.1; *Linepithema humile*: XP_012219394.1; *Lasius niger*: KMQ93879.1; *Polistes canadensis* XP_014607688.1. E2F-TDP domain and coiled coil domain are indicated with black and green lines, respectively.

**Fig 3:**
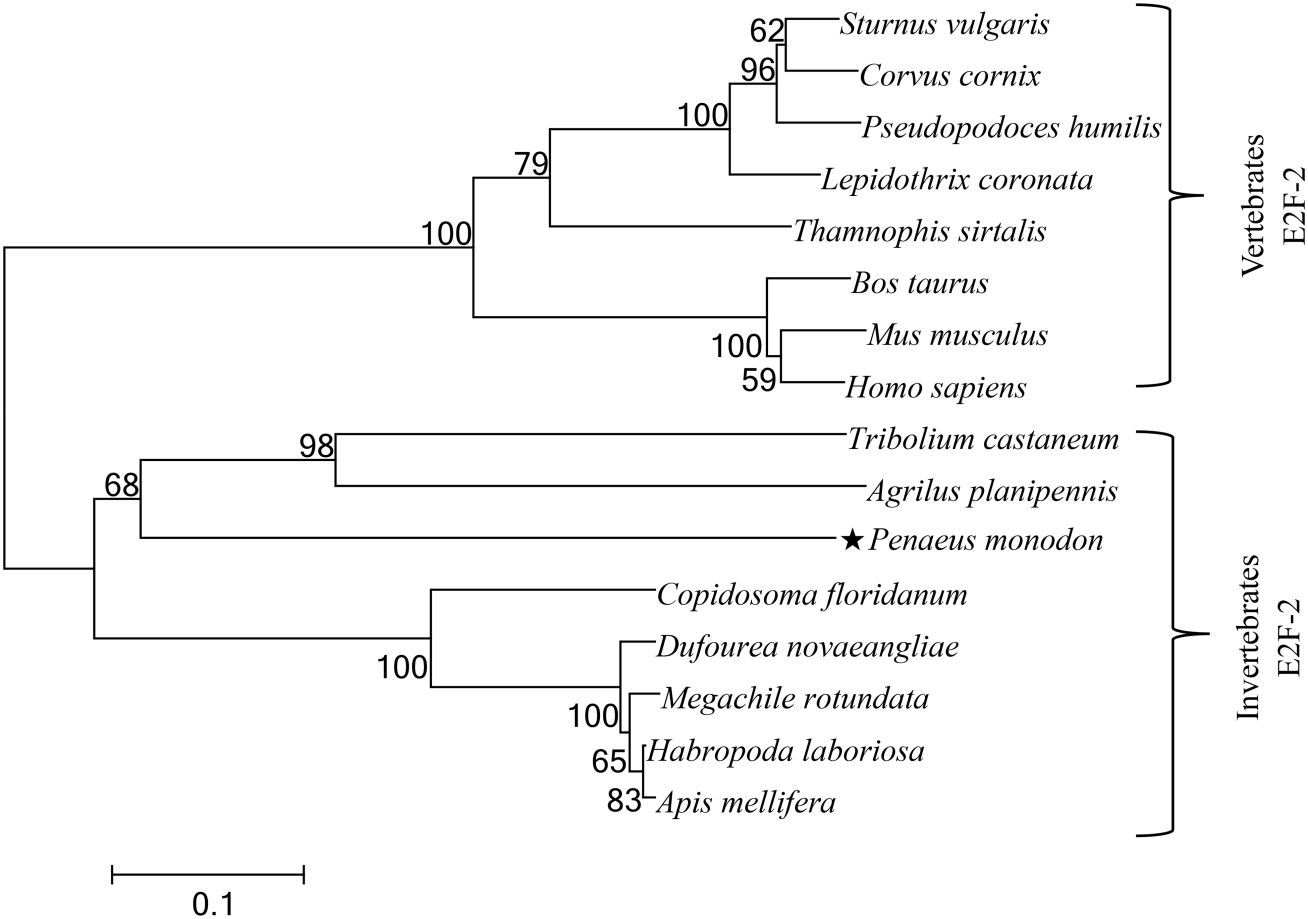
The neighbor-joining phylogenetic tree of E2F-2s based on their amino acid sequences. The confidence in each node was assessed by 2000 bootstrap replicates with Mega 5.03.

Fig 2 shows alignment of deduced aa sequences of E2F-2 genes with some known E2F-2s. Highest identity with different species was noted in functional domain of predicted aa sequence of E2F-2 genes from *P*. *monodon*. Fig 3 illustrates a dendrogram depicting evolutionary relationship based on E2F-2 protein similarity of different species. Vertebrate E2F-2 proteins are closely related to each other and converge into one subgroup, whereas *P*. *monodon* E2F-2 proteins are clustered with other invertebrate E2F-2s (See S1 Table).

### Genomic structure and upstream regulatory region of *PmE2F-2* gene

**Fig 4:**
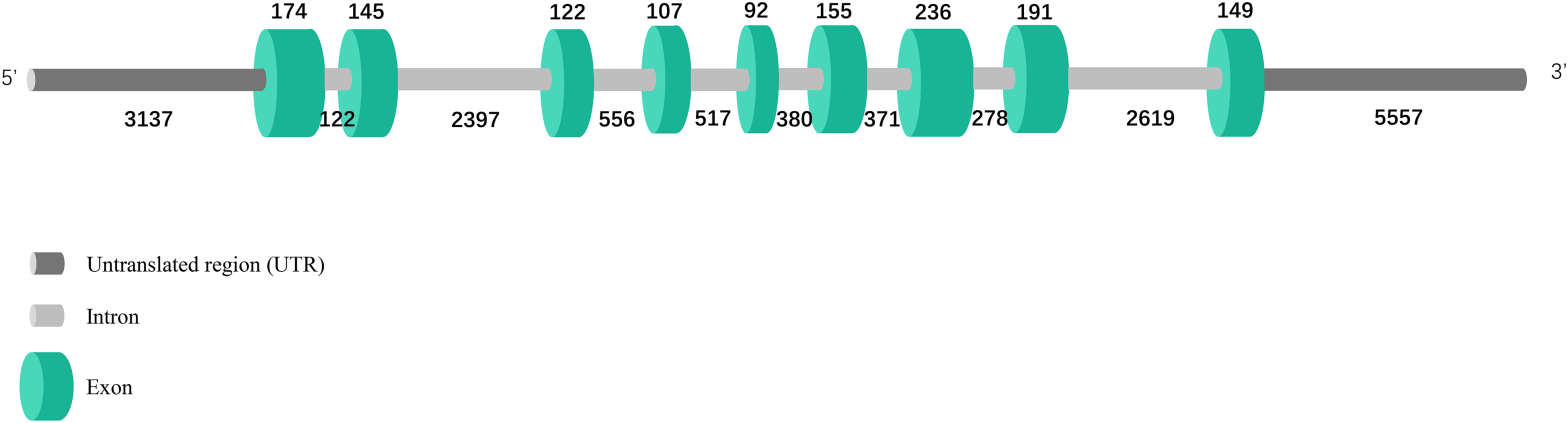
Comparison of the genomic DNA sequence encoding E2F-2 in *P*. *monodon*. Green-shaded rectangles indicate exons, gray horizontal lines represent introns, and the numbers indicate exon and intron length (in bp).

**Fig 5:**
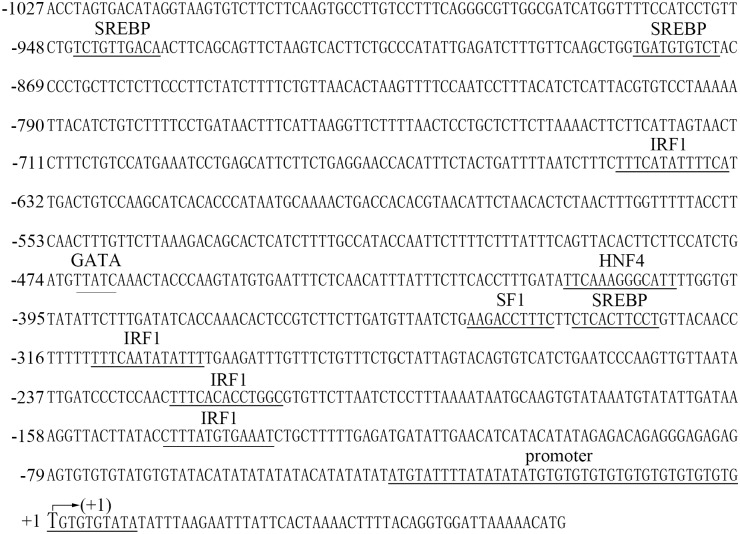
The nucleotide sequence of the *PmE2F-2* gene showing the 5’ upstream genomic sequence. The nucleotide sequence is numbered starting from the transcription start site (+1) (marked by an arrow) and proceeding as positive numbers in a 3’direction and negative numbers in the 5’direction. The putative binding sequence motifs for transcription factors, and the ribosomal promoter site, are all shown as underlined.

Complete *PmE2F-2* genomic sequence comprising 5′ upstream sequence was acquired through PCR amplification of genomic DNA and genome walking. The *PmE2F-2* gene (17,305 bp) of *P*. *monodon* contains nine exons (174, 145, 122, 107, 92, 155, 236, 191, and 149 bp), which are separated by eight introns (122, 2397, 556, 517, 380, 371, 278, and 2619 bp, respectively) (See S1 Dataset), conforming with canonical GT/AG splicing recognition rule at extreme ends of each intron (Fig 4). Some potential binding sites of important transcription factors were predicted; these sites include four interferon regulatory factor 1 (IRF1), three sterol regulatory element-binding proteins (SREBPs), one SF1, one hepatocyte nuclear factor 4, and one GATA factor binding site (Fig 5).

### Tissue distribution of *PmE2F-2* analysis

**Fig 6:**
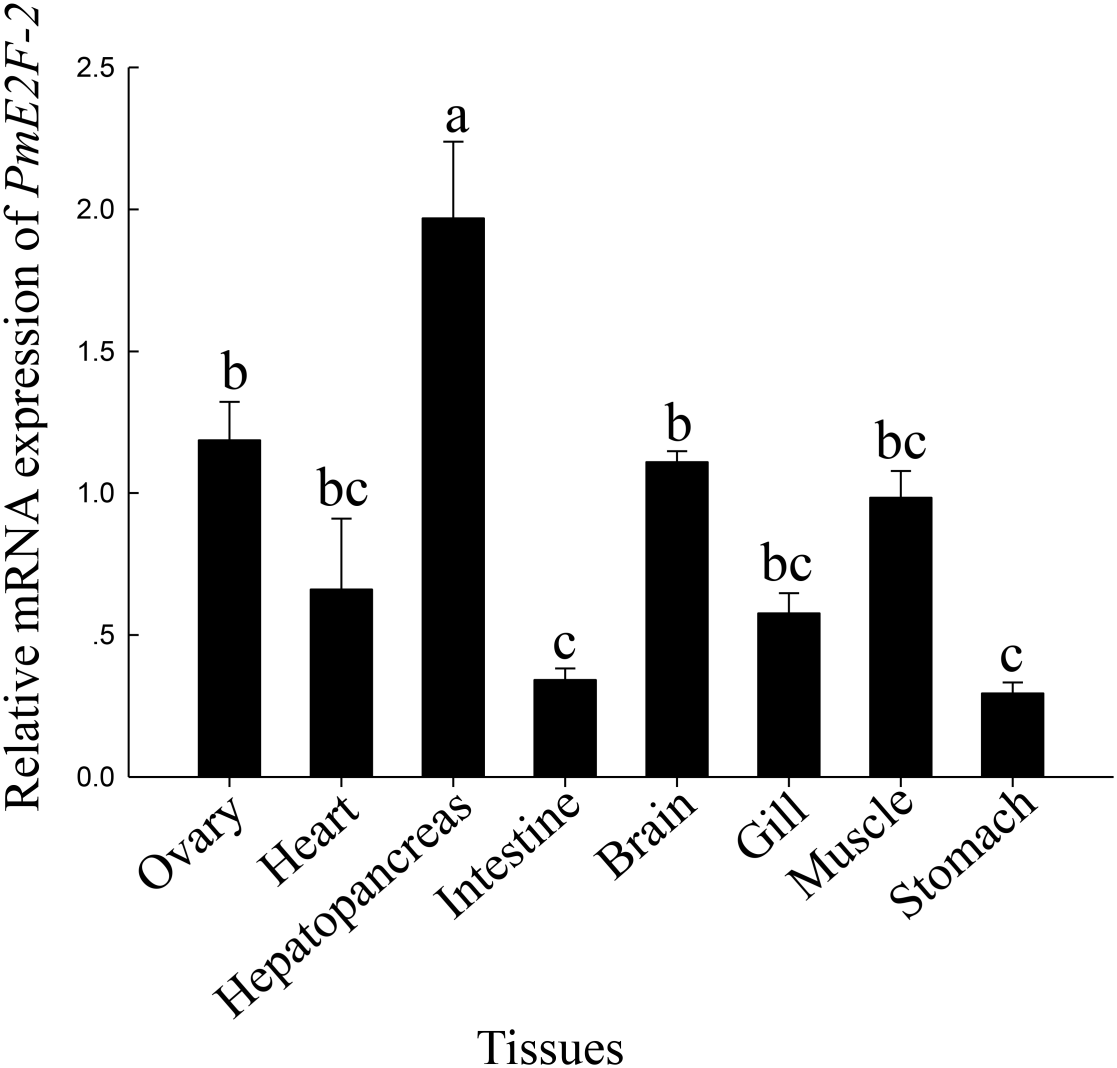
Relative expression levels of *PmE2F-2* in various tissues detected by quantitative real-time PCR analysis using *EF-1α* as an internal reference. Vertical bars represented mean ±SD (n = 3). Significant different letters above vertical bars indicate difference (*P* <0.05).

Fig 6 exhibits tissue distribution patterns of *PmE2F-2* mRNA. qRT-PCR analysis results indicated that *PmE2F-2* is widely expressed in muscles, heart, hepatopancreas, ovary, gill, brain, stomach, and intestine of *P*. *monodon*. Highest *PmE2F-2* expression levels were found in hepatopancreas, followed by the ovary.

### *PmE2F-2* mRNA expression during ovarian maturation

**Fig 7:**
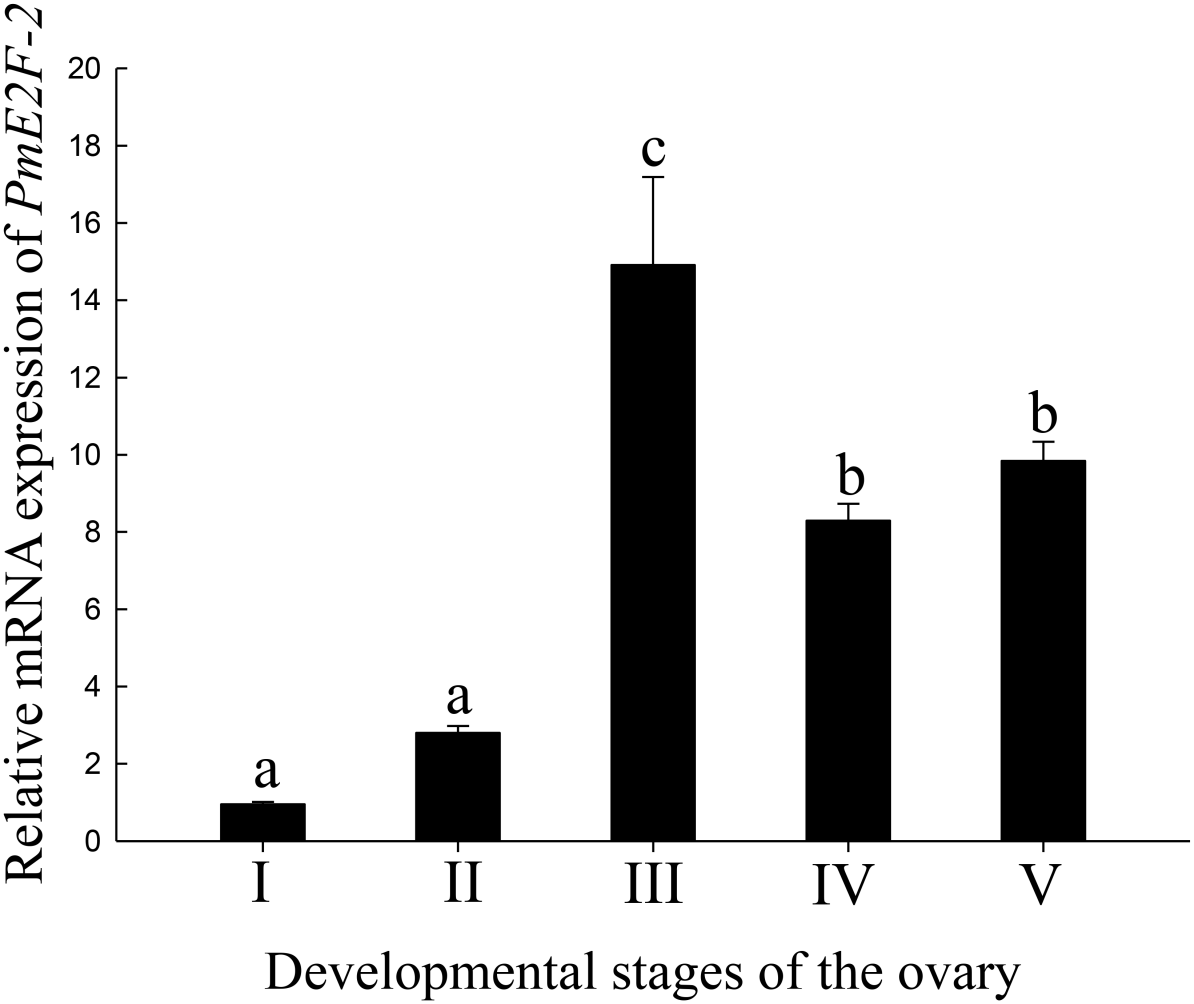
Relative expression levels of *PmE2F-2* in ovaries at different developmental stages (I ovogonium stage; II chromatin nucleolus stage; III perinucleolus stage; IV yolky stage; and V cortical rod stage).

qRT-PCR was used to detect relative *PmE2F-2* mRNA expression levels in different ovarian developmental stages of *P*. *monodon*. In ovarian developmental stages III, IV, and V, *PmE2F-2* mRNA expression levels were significantly higher than those in stages I and II, and *PmE2F-2* expression levels were highest in ovarian developmental stage III (*P* < 0.05, Fig 7).

### *PmE2F-2* mRNA expression after 5-HT challenge and eyestalk ablation

**Fig 8:**
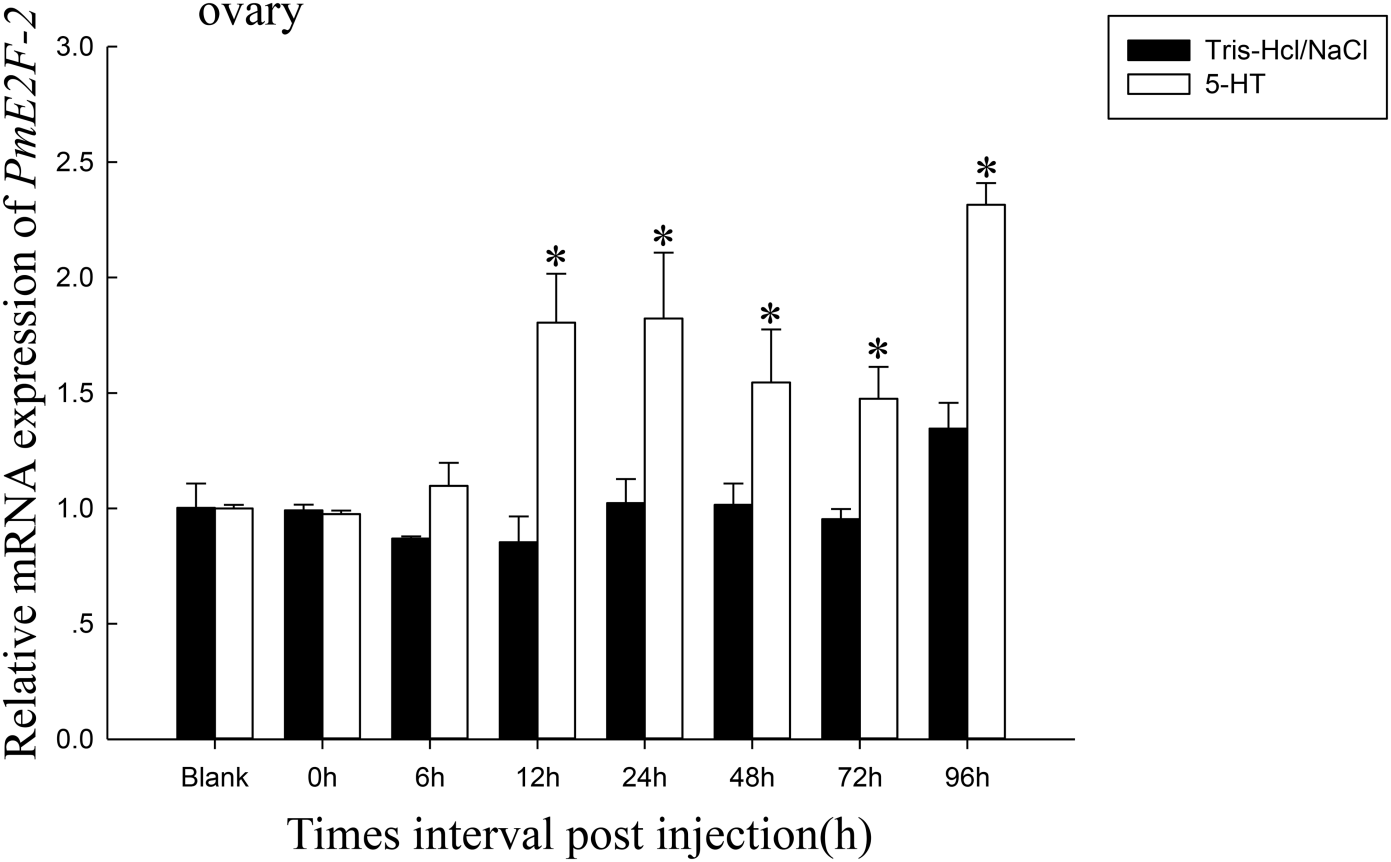
*PmE2F-2* mRNA expression profiles after stimulated by 5-HT. *PmE2F-2* mRNA relative expression level in ovary tissue post-treatment with 5-HT. Vertical bars represented the mean ± SD (n = 3). Significant different letters above vertical bars indicate difference (P < 0.05).

**Fig 9:**
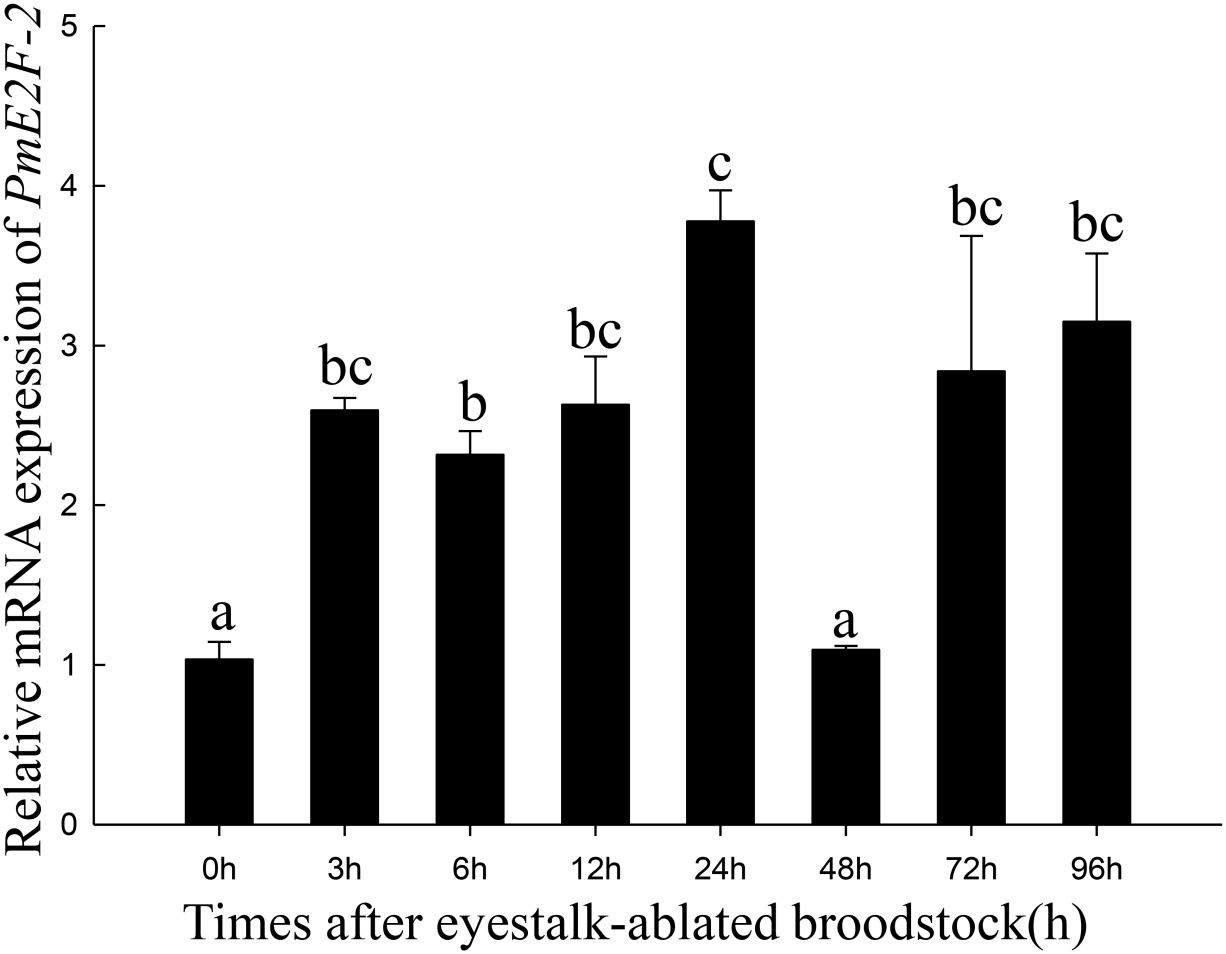
*PmE2F-2* mRNA expression profiles after eyestalk ablation. *PmE2F-2* mRNA relative expression level in ovary tissue post-treatment with eyestalk ablation. Vertical bars represented the mean ± SD (n = 3). Significant different letters above vertical bars indicate difference (P < 0.05).

We determined *PmE2F-2* expression levels after 5-HT injection in ovary of *P*. *monodon*. Results indicated that in 5-HT injected shrimp, *PmE2F-2* expression significantly increased at 12, 24, 48, 72, and 96 h compared with that in control group (Fig 8). We also investigated *PmE2F-2* expression levels after eyestalk ablation in ovaries of *P*. *monodon*. The findings showed that *PmE2F-2* expression significantly increased at 3, 6, 12, 24, 48, and 96 h compared with that at 0 h (Fig 9).

### *PmE2F-2* mRNA expression profile stimulated by dsRNA—E2F-2

**Fig 10:**
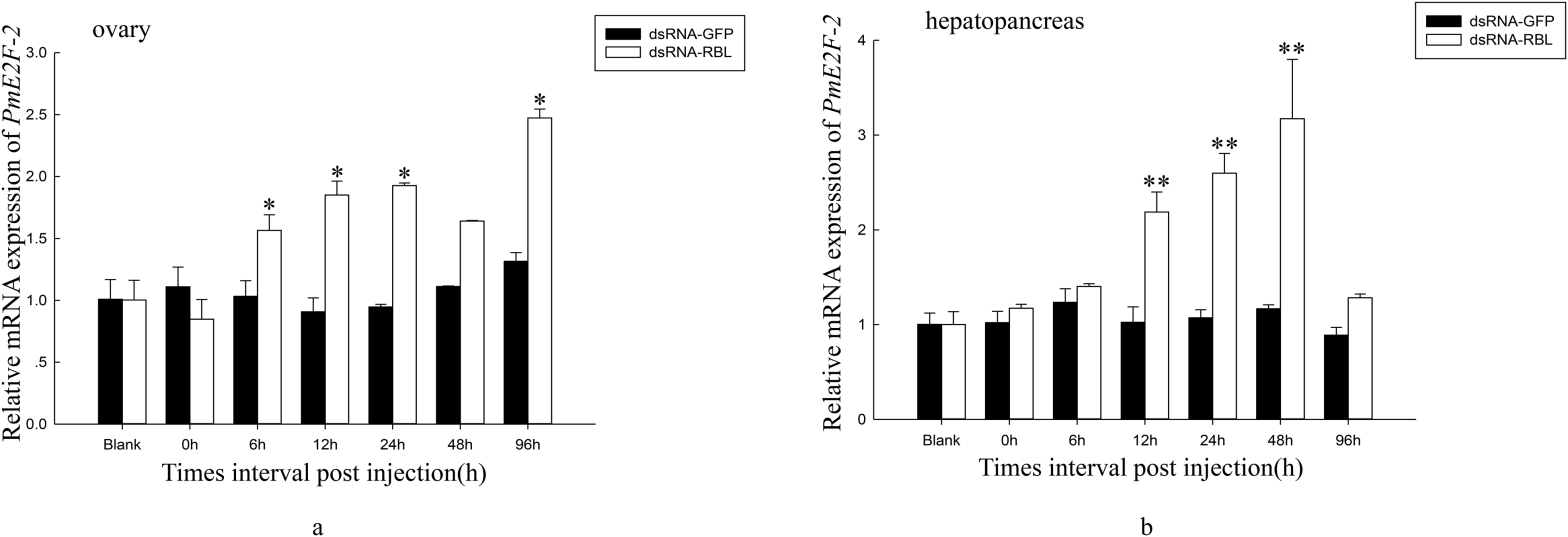
Relative expression levels of *PmE2F-2* in ovary and hepatopancreas of shrimps after treatment with dsRNA-RBL. a. Relative expression level of *PmE2F-2* in the ovary. b. Relative expression level of *PmE2F-2* in the hepatopancreas. Ovary and hepatopancreas tissues collected from shrimps injected with dsRNA-RBL were compared with respect to *PmE2F-2* mRNA expression (relative to EF-1α) using Students t-tests. Vertical bars represented mean±SD (n = 3). Significant differences from controls were indicated: **P < 0.01, *P < 0.05.

We ascertained *PmE2F-2* expression levels after dsRNA—RBL injection in ovary and hepatopancreas of *P*. *monodon* (after dsRNA-RBL injection, experimental samples were stored in our laboratory). Results demonstrated that from 6 h to 96 h, *PmE2F-2* expression in ovary was upregulated in dsRNA—RBL-injected shrimp postinjection relative to control group. However, *PmE2F-2* mRNA expression remained unchanged following dsRNA—GFP injection (Fig 10a). In dsRNA-RBL-injected shrimps, *PmE2F-2* expression in hepatopancreas was upregulated after 12–48 h; at 96 h postinjection, this expression decreased to nonsignificantly different levels compared with that of the control group (Fig 10b).

**Fig 11:**
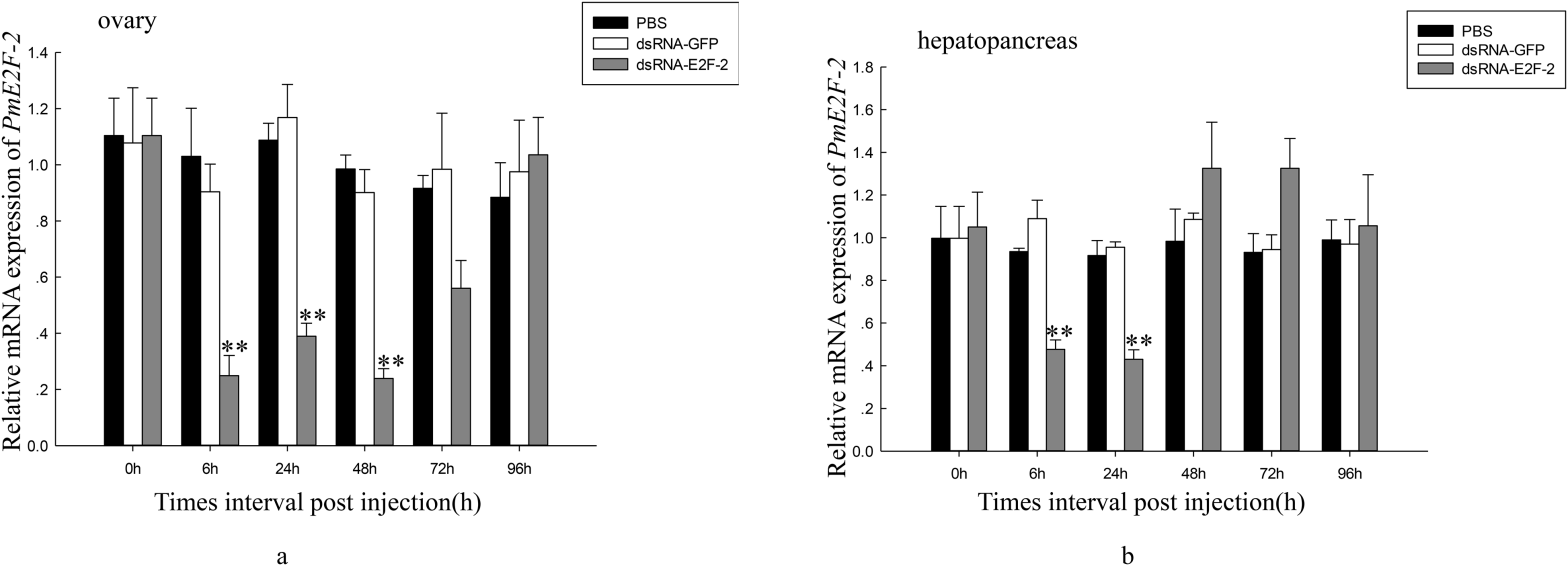
Relative expression levels of *PmE2F-2* in ovary and hepatopancreas of shrimps after treatment with dsRNA-E2F-2. a. Relative expression level of *PmE2F-2* in the ovary. b. Relative expression level of *PmE2F-2* in the hepatopancreas. Vertical bars represented mean±SD (n = 3). Significant differences from controls were indicated: **P < 0.01, *P < 0.05.

We evaluated their expression levels in ovary and hepatopancreas of *P*. *monodon* through qRT-PCR assays to determine effects of dsRNA—E2F-2 on *PmE2F-2* gene expression. The findings indicate that in ovaries and hepatopancreas, *PmE2F-2* expression levels were significantly knocked down 6 h after dsRNA—E2F-2 injection. *PmE2F-2* expression levels were inhibited in ovaries and hepatopancreas of dsRNA—E2F-2-injected shrimp after 6–48 h; this expression increased to nonsignificantly different levels compared with that of the control group. *PmE2F-2* mRNA expression remained unchanged after dsRNA—GFP injection (Fig 11).

**Fig 12:**
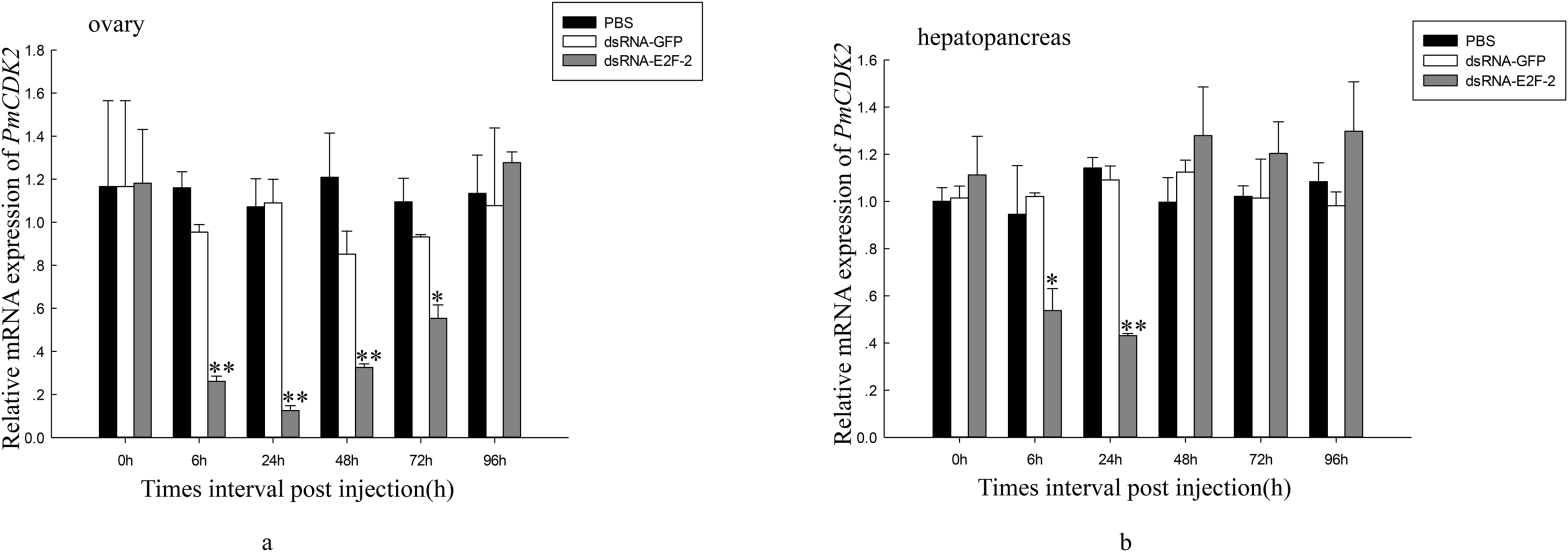
Relative expression levels of *PmCDK2* in ovary and hepatopancreas of shrimps after treatment with dsRNA-E2F-2. a. Relative expression level of *PmCDK2* in the ovary. b. Relative expression level of *PmCDK2* in the hepatopancreas. Vertical bars represented mean±SD (n = 3). Significant differences from controls were indicated: **P < 0.01, *P < 0.05.

**Fig 13:**
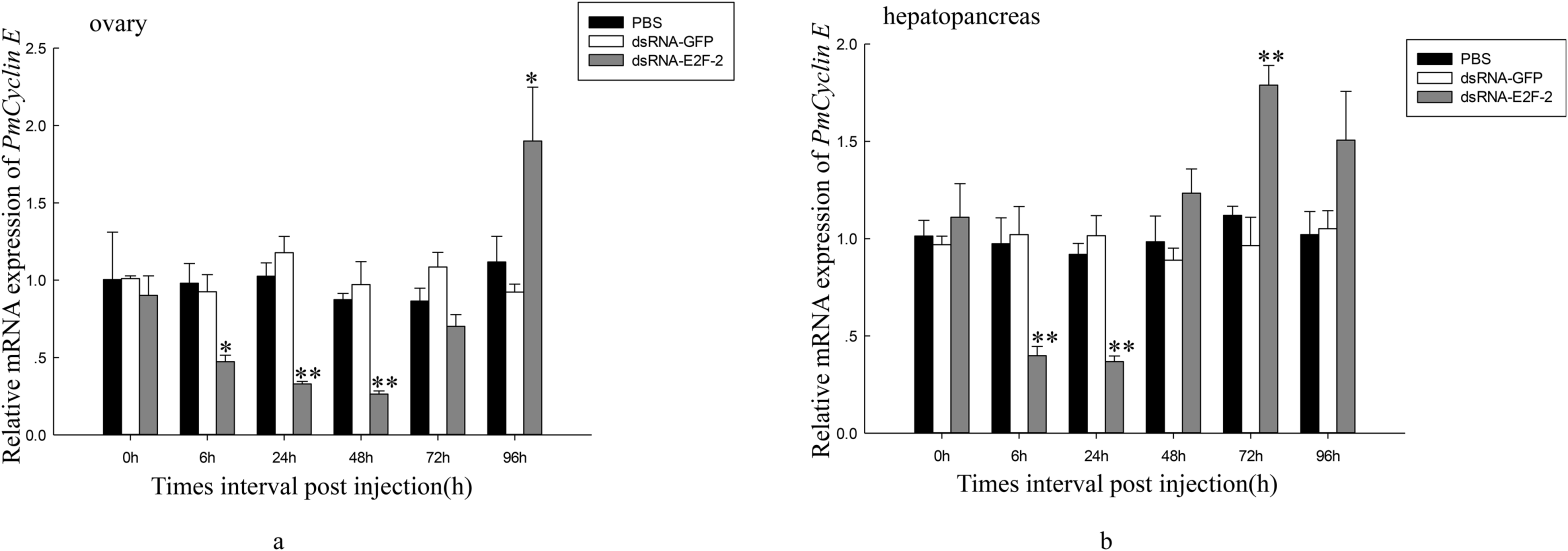
Relative expression levels of *PmCycin E* in ovary and hepatopancreas of shrimps after treatment with dsRNA-E2F-2. a. Relative expression level of *PmCycin E* in the ovary. b. Relative expression level of *PmCycin E* in the hepatopancreas. Vertical bars represented mean±SD (n = 3). Significant differences from controls were indicated: **P < 0.01, *P < 0.05.

We also investigated *PmCDK2* and *PmCyclin E* expression levels after dsRNA—E2F-2 injection in ovaries and hepatopancreas of *P*. *monodon*. After 6 h, *PmCDK2* expression was downregulated in ovaries of dsRNA—E2F-2-injected shrimp; after 96 h, this expression increased to nonsignificantly different levels compared with that of the control group (Fig 12a). Compared with that of the control group, *PmCDK2* expression was significantly downregulated in hepatopancreas of dsRNA—E2F-2-injected shrimp 6–24 h after injection (Fig 12b). *PmCyclin E* expression was downregulated in ovaries of dsRNA—E2F-2-injected shrimp 6 h after injection; after 72, this expression increased h to nonsignificantly different levels compared with that of the control group (Fig 13a). In hepatopancreas of dsRNA—E2F-2-injected shrimp, *PmCyclin E* expression was downregulated 6 h after injection, and it was increased to nonsignificantly different levels after 48 h compared with that of the control group (Fig 13b).

### In situ hybridization detection of PmE2F-2 expression

**Fig 14:**
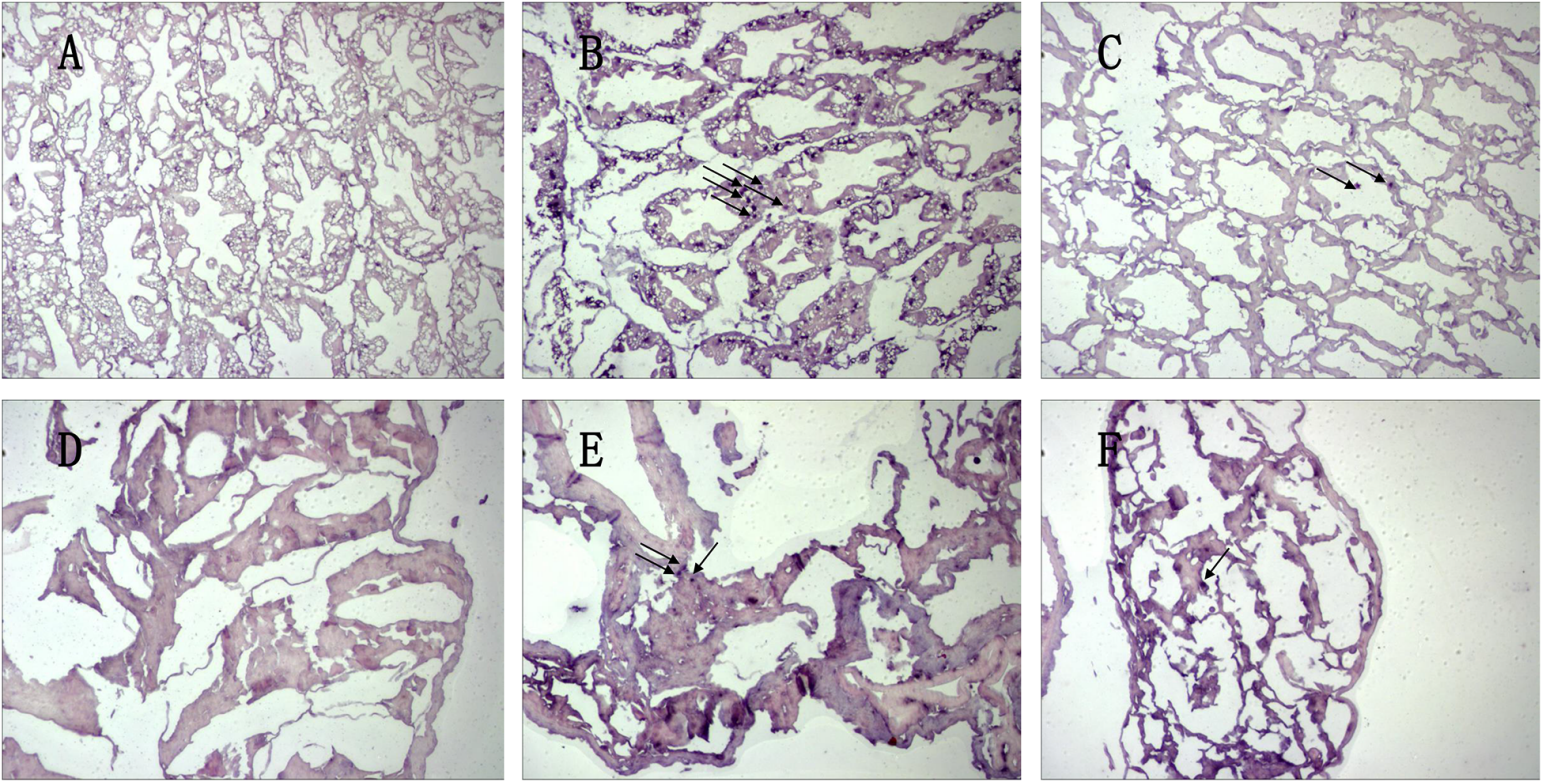
In situ detection of PmE2F-2 hybridization. The ovary and hepatopancreas were collected after dsRNA-E2F-2 injection. Nuclei were stained; the blue points indicate positive reactions (indicated by arrows). B and C represented the collected ovaries after dsRNA-GFP and dsRNA-RBL injections, respectively. A represented the negative control. E and F represented the collected hepatopancreas after dsRNA-GFP and dsRNA-RBL injections, respectively. D represented the negative control. Scalebar = 30.

Ovarian and hepatopancreatic tissues were selected and subjected to in situ hybridization analysis to determine *PmE2F-2* expression site and expression level. Several positive signals were detected in both ovarian and hepatopancreatic tissues following dsRNA—GFP injection (Fig 14B and 14E), whereas few positive signals were detected after dsRNA—E2F-2 injection (Fig 14C and 14F). Positive signals were not detected in negative control (Fig 14A and 14D). Based on positive signal quantities, *PmE2F-2* expressions were significantly knocked down in ovaries and hepatopancreas after dsRNA—E2F-2 injection compared with that following dsRNA—GFP injection. These results were consistent with those of qRT-PCR test.

### GSI detection of ovarian development

**Fig 15:**
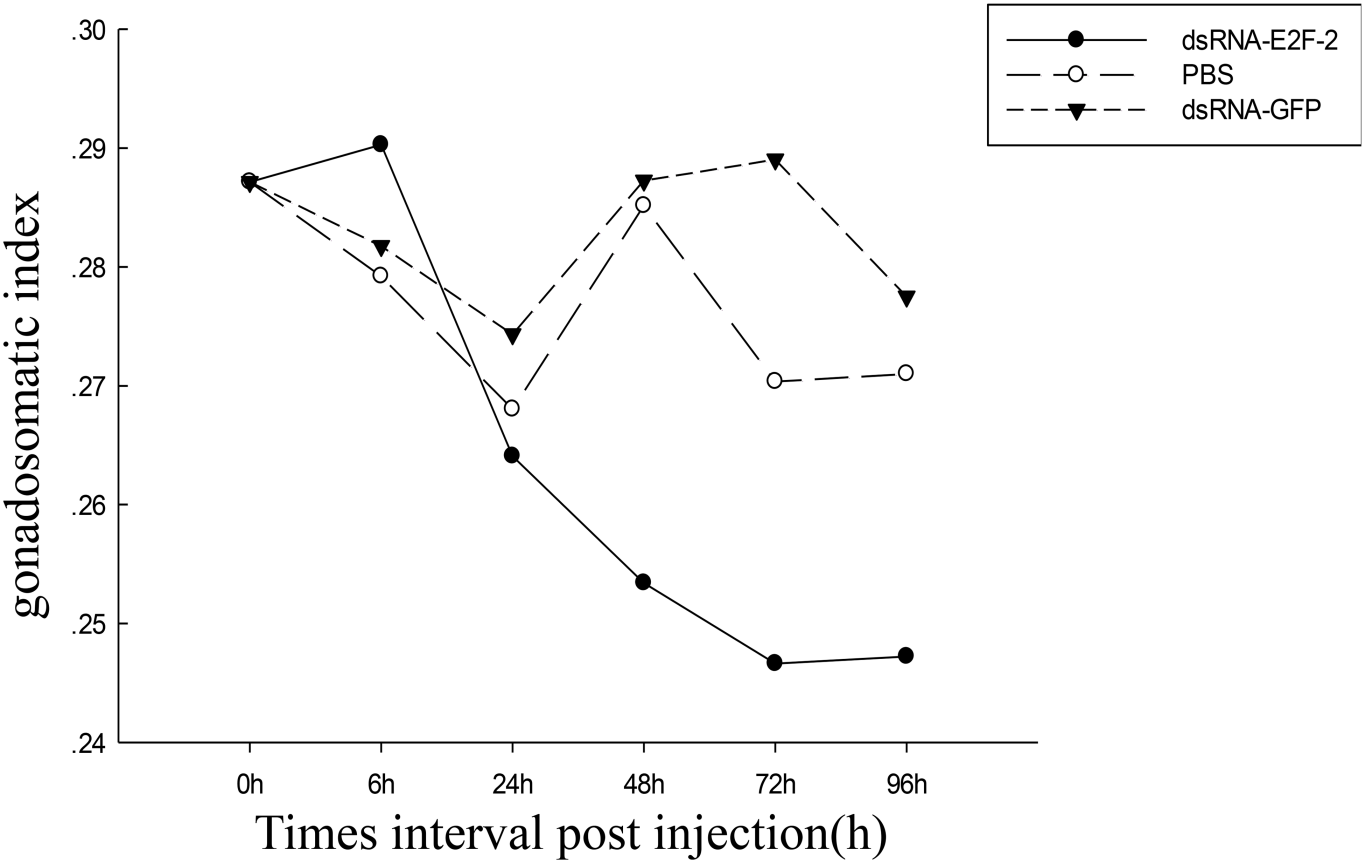
The gonadosomatic index (GSI, ovarian weight/body weight × 100) of shrimp after dsRNA-GFP-, dsRNA-E2F-2- and PBS injection.

To determine the effects of *PmE2F-2* gene on ovarian development, we measured ovary weight and body weight of *P*. *monodon*. After dsRNA—GFP, dsRNA—E2F-2, and PBS injections, GSI (ovarian weight/body weight × 100) of each shrimp was calculated. After dsRNA—E2F-2 injection, GSIs of shrimp were significantly lower than those after dsRNA—GFP and PBS injections (Fig 15).

## Discussion

E2F-2 is a multifunctional regulator involved in several key cellular processes, such as cell cycle control [6] and cell proliferation, differentiation, and apoptosis [7]. Researchers identified *E2F-2* genes from yeast to humans, but few reports investigated *E2F-2* genes from black tiger shrimp. In the present study, we first reported cloning of *E2F-2* gene homologue from *P*. *monodon*. In *P*. *monodon*, full-length cDNA sequence of *E2F-2* gene measures 3,189 bp. Vertebrate E2F-2 proteins are closely related to each other and converge into one subgroup, whereas *P*. *monodon* E2F-2 proteins are clustered with other invertebrate E2F-2s. E2F_TDP, Rb_C, and coiled-coil domains are key functional structural domains in mammalian E2F-2 and are conserved in all selected species [12]. These findings suggest that primary E2F-2 protein structure is conserved throughout evolution.

*PmE2F-2* gene of *P*. *monodon* contains nine exons, which are separated by eight introns; this observation is consistent with the report on *H*. *sapiens* E2F-2 (GenBank accession: NC_000001.11). The 5′ upstream *PmE2F-2* sequence contains various transcription regulatory factors, such as GATA, IRF1, HNF-4, SREBP, and SF-1. Further research should focus on mechanism on how these regulatory elements regulate E2F-2 gene transcription.

Tissue distributions of *PmE2F-2* were investigated, and qRT-PCR analysis indicated that *PmE2F-2* is also constitutively expressed in tissues of healthy black tiger shrimps. Highest *PmE2F-2* mRNA level was detected in hepatopancreas, followed by the ovary. Vitellogenin plays a crucial role in ovarian development; it is derived from ovaries, hepatopancreas, or adipose tissues of decapod crustaceans [24]. These results demonstrate that *PmE2F-2* may be involved in ovarian maturation.

Oocyte development involves various complicated cellular events, which temporally and spatially express differential genes to ensure proper development or storage of transcripts and proteins as maternal factors for early embryogenesis [25,26]. Stage III is the most critical period of ovarian development, and presence of oocytes stimulates accumulation of yolk substances in the cytoplasm [27]. *PmE2F-2* expression level was highest in stage III, indicating the important role of *PmE2F-2* in ovarian development. This result is consistent with findings of some studies, which showed highest *PmCyclin B* expression level in stage III [28].

Serotonin (5-HT) was reported to induce ovarian maturation and spawning in black tiger shrimp *P*. *monodon* [29]. *PmE2F-2* expression of 5-HT-injected shrimp significantly increased at 12, 24, 48, 72, and 96 h compared with that of the control group. *PmCDK7* expression level was also augmented following 5-HT injection [17]. However, studies provide limited information on 5-HT regulatory mechanisms in crustaceans. Further investigation is thus needed.

Eyestalk ablation is commonly practiced in crustaceans to induce ovarian maturation in captivity [30]. In our study, we evaluated *PmE2F-2* expression levels in ovaries of *P*. *monodon* after eyestalk ablation. Results indicated that *PmE2F-2* expression significantly increased following eyestalk ablation. This finding is similar to that of a previous study on *CDC2* in *P*. *monodon* [31] and vitellogenin in *Macrobrachium nipponense* [32], where expression level also increased after eyestalk ablation. Such result demonstrates that *PmE2F-2* may be involved in ovarian maturation.

RNAi was used to clarify latent relationship between *PmE2F-2* and ovarian development, particularly the role of *PmE2F-2* in ovarian development of *P*. *monodon*. Previously published studies described the use of RNAi in illuminating gene functions in shrimps. For example, a study reported that *Pmp53* expression knockdown indicates that *Pmp53* may significantly influence ovarian development of *P*. *monodon* [13]. Silencing of gonad-inhibiting hormone transcripts was performed to establish an efficient RNAi-based technique to stimulate gonadal development in *L*. *vannamei* [14]. In the present study, we successfully knocked down *PmE2F-2* genes in ovary and hepatopancreas via dsRNA—E2F-2 injection. *PmE2F-2* expression of dsRNA—E2F-2-injected shrimp was inhibited after 6–24 h and was then increased after 96 h to nonsignificantly different levels compared with that of the control group. These results are consistent with those of a previous report, which revealed temporary specific inhibition of gene expression through RNAi-based techniques [33]. Several studies reported *E2F-2* gene function using RNAi. RNAi-mediated E2F-2 knockdown inhibited human glioblastoma cell tumorigenicity [34]. After 6–72 h of dsRNA—E2F-2 injection, relative *PmCDK2* and *PmCyclin E* expression levels were downregulated in ovaries and hepatopancreas, revealing that *PmE2F-2* silencing can decrease *PmCDK2* and *PmCyclin E* expression level of *P*. *monodon*. Previous studies often referred to E2F-1, E2F-2, and E2F-3 as “activator” E2Fs because they transcriptionally activate E2F target genes, such as cyclin E and CDK2, that aid cell cycle regulation [10]. Rb functions as a cell cycle repressor through inhibition of E2F transcription factor activity. Hyperphosphorylated Rb releases E2F and promotes expression of genes mediating entry into the S phase [9]. We also determined *PmE2F-2* expression levels in ovaries and hepatopancreas of *P*. *monodon* following dsRNA—RBL injection. Results demonstrated that in ovaries and hepatopancreas of dsRNA—RBL-injected shrimps, *PmE2F-2* expression were upregulated after 6–96 h relative to that of control group. Dai et al. [13] reported that *PmCDK2* may be involved in vitellogenin synthesis and ovarian maturation in *P*. *monodon*. These findings suggest possible involvement of *PmE2F-2* in ovarian maturation.

We studied *PmE2F-2* expression site and expression level in ovaries and hepatopancreas via in situ hybridization. Several positive signals were detected in ovary and hepatopancreas following dsRNA—GFP injection. By contrast, only few positive signals were observed after dsRNA—E2F-2 injection. Considering the quantities of positive signals, *PmE2F-2* expression levels were significantly knocked down in ovaries and hepatopancreas after dsRNA—E2F-2 injection compared with those following dsRNA—GFP injection. These results were in agreement with those of qRT-PCR test.

In crustaceans, GSI is a gross quantitative indicator of gonad condition and is the simplest way to measure changes in size and weight of this organ relative to total weight of organisms [35]. After dsRNA—E2F-2 injection, GSI of shrimp was significantly lower than those after dsRNA—GFP and PBS injections. These findings demonstrate that *PmE2F-2* may play a crucial role in ovarian development.

In conclusion, *PmE2F-2* cDNA sequences were cloned and identified. Complete genomic sequence of *PmE2F-2* from *P*. *monodon* contains nine exons, which are separated by eight introns. *PmE2F-2* is highly expressed in hepatopancreas and ovaries and during stage III ovarian development of *P*. *monodon*. *PmE2F-2* expression levels significantly increased in ovaries of *P*. *monodon* following 5-HT injection and eyestalk ablation. *PmE2F-2*, *PmCDK2*, and *PmCyclin E* expression levels were determined after dsRNA—E2F-2 injection to examine their relationship with one another. We investigated PmE2F-2 expression localization and levels in ovaries and hepatopancreas through in situ hybridization, which revealed consistent results with those of qRT-PCR. After dsRNA—E2F-2-injection, GSI of shrimp was considerably lower compared with those after dsRNA—GFP and PBS injections. Findings of this study can improve our understanding of the molecular mechanisms underlying ovarian development in shrimps.

## Supporting information

S1 Table Dates used in the phylogenetic analysis. (PDF)

S1 Dataset Complete genomic sequence of *PmE2F-2* gene. (PDF)
