# Supplementary material for: Genomic structure, expression pattern, and functional characterization of transcription factor E2F-2 from black tiger shrimp (Penaeus monodon)
Source: PLoS One. 2017 May 30;12(5):e0177420. doi: 10.1371/journal.pone.0177420 (PMC5448752; doi:10.1371/journal.pone.0177420)
Supplement: S1 Table — (PDF) [file pone.0177420.s001.pdf]

Species	Gene name	GenBank No.
Thamnophis sirtalis	Transcription factor E2F2-like	XP_013926824.1
Habropoda laboriosa	Transcription factor E2F2-like	XP_017797358.1
Apis mellifera	Transcription factor E2F2	XP_006561712.1
Megachile rotundata	Transcription factor E2F2	XP_003699580.1
Tribolium castaneum	Transcription factor E2F2	XP_008200394.1
Copidosoma floridanum	Transcription factor E2F2	XP_014210403.1
Lepidothrix coronata	Transcription factor E2F2	XP_017686548.1
Pseudopodoces humilis	Transcription factor E2F2	XP_005529715.1
Sturnus vulgaris	Transcription factor E2F2	XP_014745997.1
Corvus cornix	Transcription factor E2F2	XP_010406471.1
Agrilus planipennis	Transcription factor E2F2-like	XP_018323436.1
Dufourea novaeangliae	Transcription factor E2F2-like	XP_015436200.1
Mus musculus	Transcription factor E2F2	AAH62101.1
Bos taurus	Transcription factor E2F2	XP_002685823.2
Homo sapiens	Transcription factor E2F2	AAA16890.1
