# Supplementary material for: Genomic structure, expression pattern, and functional characterization of transcription factor E2F-2 from black tiger shrimp (Penaeus monodon)
Source: PLoS One. 2017 May 30;12(5):e0177420. doi: 10.1371/journal.pone.0177420 (PMC5448752; doi:10.1371/journal.pone.0177420)
Supplement: S1 Dataset — (PDF) [file pone.0177420.s002.pdf]

Complete genomic sequence of Transcription factor E2F-2

TTCCTGACTTTTTAAAATTCTTCAAAGAGAATAAATTTATCGTGAACTCACAATTTTCTAAATTCATAAACTCATACGT
CCCTAACTTTATAAATTCCCACACTCGTAAACTCACAGATTATTTTTTCAAAATTCATGAACTCAACAAATTAAATTTT
CTTGAACTATTAAAATGATGAACACATAAGATCATGATTAATCAAACTTATGAACTCATAAATTCCAAACTCGTAACTT
GCTAAACTAAAAAAATATATATATTTTTTTAAACCTCAAAAACTTATAAATACATAAACTCATAACCATACTCATAAAT
TCAAGAGAAGAGTTTTTTTTTCTGCACCGTGCATAATGGCAGCAGCAGTTGCAACGTAGTTTCATTGAGGGTTTACAAA
CGGCAAAAGGAACAGCAATTTCCTGAATTATGTTGGGGTGCGTGAAGCCGCCGGTTTAAGTGCAAAACGGCAAATGTTT
TCCCCTACAATATACAGGACCGTTGCATCCGTTATGATACTCTAAAGTAATGTTTTGTTTATTTGTTTATATATTTTGT
TTATATCTGTGTCTTCATTTATTTTTTTATTTTATTTTCTCTGAGTTCGGTGAAGCGGTGTACAGGCAGCCGAGGTTTA
TTTGTCTGAGAGTTAGAAGTAACTTCCATTGCATCGGGACGTGTTTATAGTGTGTGTTTTGTGGTAGACATAGTTTGAT
TTGATAGAAAGATAGATCGTGGATAATTATACCTAATGCGGTTACTTTTGCGTTGAGATATGGCAGTATAAGCTGTCTG
TTAGCATTCATAGTATTCGGTTTATTTATCTAGCGGAGTATCATGGCTGGTAAAAATGAATGCACCATAAGGGCTTCAT
ACACACCCAGAGACAAACACACACACACACATAGCACAACACACACACATAGCACAACACACACATAGCACAACACACA
CACATAGCCAACACACACACATAGCACAACACACACACATAGCACAACACACACACACAGCACAGCACACACACACAGC
ACAGCACATATGCACCCACAACAAAAAAGCACACACACACATACGTGAACATTAATCCATAAGCCCACTTAACACACAT
GAAGAAAGCTGGATGAAAATTACATCCTAGTAATGGCAGGAAGAAATTCTAGGCCTATGTGAATATTAGAACATGTGAG
TGGATATATTCATGGCAAATGTTTGAAAGTATTACATTATTTTGTATTTTTTAAAATATTCATTTTTCAATTTGTCATA
AAACAATCTGAATATTCAAACATAAAAATGTAAATGTTTCTATTGAATATGCTATTCCATGAACATGTTACAGTACTAA
AATTTAAGCAAGCTTTTTTGCACTATTCTGTCAAAGTAGTCTTTTTATGTTTATTCATTCTTTTTTAGTTGACAACTGC
CAGTTGTAAGTGAATACAAAGTAGTGATTCGTAATTACAAGCTAGAGAATGATTGTCCAGAACCTTAGCACAGACCAAT
CTTGCAATCATGTTGACTGACTGAGCCAGTTCCTGCTTCTTTGACTTATGCAATACTATTATGATATATAATGGTATAA
GAAATTGAGTTGCAGTACATGGTGGAGAGGTCCACGGCTGCTCAAACATGAGCATACCGTTATTGATGATGATGAACCA
TATGCATGTCATAGGCACAAAGGAATTTCGAAATAGTCATACTTGGCAGTAACACCAGTCAGTTAAAATCATACCTATT
ATCTGGCGAAGGATGGTGGAGAGGTCCACGGCTGCTCAAACATGAGCATACCGTTATTTATGATGATGAACCATATGCA
TGTCATAGGCACAAAGGAATCTCGAAATAGTCATACTTGGCAGTAACACCAGTTTAAAATCATACCTATTAACTCTTCT
TTTTTCCTTAATTTATGCACTAAAGATAATTGGCCAGCATTATTGATATGATACTTGGCACTAAAGATAATTGGCCAGC
ATTATTGATATGATACTTTATCTTTACAGTGTGAGGGAACTGAGTGGTGCTAATATTGCCCAGTTGTGGGCAATTGTAC
ACCTAGTGACATAGGTAAGTGTCTTCTTCAAGTGCCTTGTCCTTTCAGGGCGTTGGCGATCATGGTTTTCCATCCTGTT
CTGTCTGTTGACAACTTCAGCAGTTCTAAGTCACTTCTGCCCATATTGAGATCTTTGTTCAAGCTGGTGATGTGTCTAC
CCCTGCTTCTCTTCCCTTCTATCTTTTCTGTTAACACTAAGTTTTCCAATCCTTTACATCTCATTACGTGTCCTAAAAA
TTACATCTGTCTTTTCCTGATAACTTTCATTAAGGTTCTTTTAACTCCTGCTCTTCTTAAAACTTCTTCATTAGTAACT
CTTTCTGTCCATGAAATCCTGAGCATTCTTCTGAGGAACCACATTTCTACTGATTTTAATCTTTCTTTCATATTTTCAT
TGACTGTCCAAGCATCACACCCATAATGCAAAACTGACCACACGTAACATTCTAACACTCTAACTTTGGTTTTTACCTT
CAACTTTGTTCTTAAAGACAGCACTCATCTTTTGCCATACCAATTCTTTTCTTTATTTCAGTTACACTTCTTCCATCTG
ATGTTATCAAACTACCCAAGTATGTGAATTTCTCAACATTTATTTCTTCACCTTTGATATTCAAAGGGCATTTTGGTGT
TATATTCTTTGATATCACCAAACACTCCGTCTTCTTGATGTTAATCTGAAGACCTTTCTTCTCACTTCCTGTTACAACC
TTTTTTTTCAATATATTTTGAAGATTTGTTTCTGTTTCTGCTATTAGTACAGTGTCATCTGAATCCCAAGTTGTTAATA
TTGATCCCTCCAACTTTCACACCTGGCGTGTTCTTAATCTCCTTTAAAATAATGCAAGTGTATAAATGTATATTGATAA
AGGTTACTTATACCTTTATGTGAAATCTGCTTTTTGAGATGATATTGAACATCATACATATAGAGACAGAGGGAGAGAG
AGTGTGTGTATGTGTATACATATATATATACATATATATATGTATTTTATATATATGTGTGTGTGTGTGTGTGTGTGTG
TGTGTGTATATATTTAAGAATTTATTCACTAAAACTTTTACAGGTGGATTAAAAACATGGATAGCGTTGGGACCCCTTC
CTCGGGGGGGTTGTCATTGGTTGGGGGTACTGTCCTCAGTGTCTGTGATGGGATGACCTACACACAACTGCTTGACCAT
GGCTATGGCTTAACCCCCATTACACCTAGCAACTCCAAAGAGACTCCCACAGTAACTCCCGGACGCACACAAGTAAGAT

ATCCTGGATCTTATTCTGTTTGTTTTTGATTTGTTTTTAAATGTCATATTTGGTATATAGAGTGTTTGTATTTTGAGAA
GTAGAAGCATGAATGTCTTCTGATGAATGTTTGCAGAGTGTAAAACGCCGGTTGGTACTAGAGGAAGGGGGCGTTGATG
GGGAGGGGTTCAGAACACCCACGAAGACTGCCAGACGTGCTAGGCAGAAGTCAGTCTCAGCCACACACTACACTCCCTC
ACCGTCCAAGGGGAAAACTCCAGGTATGATAATAGTATTTTGATATCACTTTTTTATGAAATTTAGGGCTTAGCTTGTA
TGTTGAGTTTTTAGCAACTCCATTATCAAATATCCTTTTCTCATTTATATTTTAATTGTAACTATACTGTACATGATAC
ACAAGTTTTTAGATGTGTGTAGGTTTAATGTAAAATCTATATTTACAGGAGAGAAATTAGTAACAAAAATTATTTGGTA
ATCTTGTATAAGAGTTATGTATTATGCTTTGCTAAAGTGCAAGCAGACATGGTGAATGTTGTTGTAATTACTTGAGGCC
ACTTTAGTTGCCAACAATACCTGCATTTAGTATTTAGTAATTTTTTATTCTGGATTAGTGAATATTGGCAAAATTGCTA
ATATGACATAATTTCTACCAACCCAGTAGGTCAAGTAATGGATACTCTCTCCATTGTATAAATATAATCTTTTAAATGC
CTACAAACTTGACCATGTTAAACATACTCTTGTTTTACTGGTTGCAAATCTTGTCTGACTTGGGTCCTAGGTGTCCCTT
TTAGTGCACTGTACTTTGTCTCACAGTATGAGAAACACACATGCACACACACACACACAAATCATGAAGTGGTCATATC
TCCAGTAAACACAGCACTAGTGGTTTATCCTCCCTGACTGCCTCCTAAAGGCCTCAAAGTGTGTTCAGAACTAGATAAG
ATGTTGTGCATGTCCTCTGTTGACAGAACTAGCTGTCATTGAACTCCTAAGTTCTTTGTACACCACGTTCATGTTGACA
ATGGCTGTGTCCAGAAGGTAGAAGAATTTCTTGTACTAATACCTGAGAATACTGGTCATGCAGCTTATGCCCATGGCAT
AAGAGAAACCATCTCAGTGGTGTAGAACATTGATAACATCCATCTGCAGACAAAGTATGGGTTATAGATTTGCTCTAGT
AGTTCACAGTCATGACCCTTTGCATTGGTGGTCCATCAGCATCTGTTTTCAGTTAGTTCACACCATACTGACATCATTG
GTAGAATGGAACTGCAGGTAGTGAAAGAGGATTGGAGATGCACACAGTTGTCAGTATGTAACATGTACCCCTTTCCATA
GAGATTAGTAGCTTCTATCAAGGCAACCTTCATGCTGACAGGAATTTCTTTATGATTATGTTCCATGGAATCCTAGAGT
GGTTGTGTGCCTGGTCTCCACTCCATTACTGTTTGTATGTCTTCATGCCCCACTGTGTCCTCTTGTTAGCATATACTGC
AGGTCATGATAGTACCACTGGAAGATGTTGATGATAAGGAAGACTTCCAGTTAGTCATTTGTTTGTTGTTCTCCATCAG
TGTCTGTGAAGTGCACGTTCATAGGTAATGCATAGAAGAGAAGAGAGAATTTAAAGATCTTGTTTGTAAGACTGCATAA
CCATGCATATGAATAAACATTCAGAATAAGTAATGGGAACTTGTTGCTCCTTGGCAATTATGATCATGAGATTATCTGT
CATGAAGCAGTATTCAGGTTCTTCCTCATATTATCCCTTTGACTATGACTAACCCCCCCTTCATAAATGGACCTCAGGG
TTCACAGTGAATACAGAGGGTTTTGATATCCCAATATTCTATGGAAGGAGACAATTTATGGTAACAATTAGTCTCCCTG
GGCCAGCAACTCAAGCTAGCACTCTAGCTTTAGGTGGGAGGTTACCTAGGACACTCCTCAGACAAAAACAGCAGCCATT
TGACTGAAGCAGTTGGAAAAGAGGAATCTCCAACTTGCCACAGAGACATCAATGGGAGTTACCATATCCAAGGGGTTAA
AACATCAAAATGCTTTTAGATGTGAAAAATAAGGCTAAGGAAATTAAAAGGAAATTTCCAGTACCAGTAATAGTTTACA
CTGAGCAAATAACATTAACCCAGTGGTAATAGGTTTTCCTGCACATACGTAGGAGTGCGAACATAGTCAGCCACCCTAG
TGTAAAAAGAGATGCATAGGAAACACATTAACTCTTCTCTTTGCATGCCTGGCTGCAGCATACTCAGTTTTCTAGTAAC
TAAAACCAAAACAAAGATTTAATCCAGCTTTTGAGGGGCTGTAGCTCCTGAATGACAAAACTGGAGCAAATCCAGTGAA
CAACTGATAAGGACACACACCTCGGAGTAGAGTCACCGTCAGGATTAACCCAATGTGCCCGGGCCTTTTCAGCGCTCAT
GTGGTCTGCCAAACGGTTATATCTGTTTGGGCCACATCTGTAGAGATGCCACCCGTGGCTCAGCACTATGCTGCCATGG
CATCATGGTAGATGCCACCCGCAAACAAGGGGTTAATGGTGCTGCAATGCAGTTTCCAAATGTATCATGAGATGCCTAA
AGCATCTTAAATTTAACAATTAAATGTAATGCACTGATTGTTTATTTTAGCCGTCCCTCCGGCACCATCCCCGGGTAAG
TCCTCAAGATACGACACCTCCCTTGGGCTCCTTACAAAACGCTTTGTTGACCTACTCCAATCGGCACCTGATGGCACTG
TTGATCTCAATAGGGTAAGGATTCAGAGTTAGTAGATTTGAAGTAGCATTATACTAGGCTGTTTGTAATTATACAGAAT
GACAAAAAATTTAGATTGCTATTCTGAAAGAATTACTAATAAGTAGTTTCAAAAAGAAAGTAAACTATTTTGTTATTTT
ATGTCAGGTTGTGTTTGTCAAGTTACAGTGGATATCATTGTTTTACTTTATTTTTTATTTTCATTATATAAACAAACAT
TTAAGTTTTATCGTTTTTGTAGTTTAGAGATCAGATACACAAAAAAGTAGTGCTATTAATGCACTCCTTATTTTGTTCA
TGAAATAGTAAGTGGTGACATAGGTGACTGAGGTTAGCATCTGGTCACCCAGACTAAAAATTACACATGTGGAAATTTC
CTTATGTGTGCCGCATGTCTGGAAAATTAAACTTGGATTTGTAACACTTGCTTTGCCTTAAAATAATGCAAAGAAGGTT
ACAGATTGAACATTTATTTATAACCAGGATTGTAATATGCTGCTTTTTCTTTCAGCATAAGGTATGATCATATTGTCTT
GTTTTGTTTGTTTACAGGCCTCTGACAAGCTGTCTGTTCAGAAGAGGAGAATATATGATATCACAAACGTCTTAGAGGG
AATTGGTCTAGTCAACAAAAAGTCCAAAAATAATGTTCAGTGGCTGTAAGTAGAAAATGGTAAACTATTTTGTTGTAAC

CCTCAGATTCATTAACAAGCAACTACACTTCCCAGCTCTTTTCTGATTTTTTTTTATGGGATTGCCAGTTATTTAATAA
CAGATATTTCCTCAAAACAAGCTGTGCTTGAAGTGTACTGTTTGCAATTTGTAGAATAGGTTTTTTGTAAAGATGTCTG
GTCATAATGTTTGTATGTAACACCTACAAATAAATTTTTGTCCTGCTGTTTCCTCCATATTTCCTCCACCTCCTTCATT
TAATTTCTTGCTGCTGCTCACCTGTTAAAGTAGAAAATGGGCTCCTGTATTTCATTAGGTAATGTCTCCTGTAGAATGA
TGCAGTCAGAAAGTCTATATCTGTAAAAGTCTTGGATGGAACTAATAGACTTCTTATTGTCATGGGGTACAAGGAAACC
TGTTTTTATAAATATACCAAGAAAACAAAGCACTACCTATAGAGATGCAAAAAAATAAAATTATGTACTAATATTTCTG
TATTTTCAGAGCTTCACGGATGAGCAGTCAAAATTTAGAGGGTGATGTTGAACATCTTGCTTCCAAAGAAAATGAACTT
GACAGATTAATAGAACAAGCAGGTTTGTTAAATATAATTGCTGTATATACCTGATTTCTTTTCTTAGTATAGAATTTCC
ATTACCATATGCCCAGGCTAATAATTACATACTTGATTGGTACCAAGTATGTGCCGCATGCCTGGAATATTAATTGGGA
CTGCAGCACACTTCATTTTTCCGTCTGGGGGGAAATGATGTGCTAAAGTTTCTACAGTTTTACATAAATTTGAGAATTT
TTAAGGTTGTTAGTTTGGTTGAAGCAATTTATTTTGCTGTATGTTCTTACAGTTTTAAAGGTATTTGTTCTTTCAGTTA
TGAATTTATAGATGTTGCAAGTTTTTCAAAGATTTCAATTTTTCATGGCTGCACTCAGAGTACATGTTAAAATTTTCTT
TCAACAGAGAGAGACCTCCTGCAGATGAGTCAGGACAAAAGATATGCCTACATTACATACCATGATCTTCACACCATCC
GATATTATAAAGACAAAACTGTGTTTGCTGTGAAAGCGCCACCTGGAACACAACTACAGGTCCCACAAGAGGTCAAAGA
GCAGGTAAATTTTTGCAAAGTTGCTTAATGAATATTGTCATAAACTTTATTCCAAATACTGTTTAATGTTTACATTTGA
GAAGGTGAATTGTGTGATATAAATGACTTTGGTATGTTATGGTGGTTGTAATTTTTCAGGGTTATAAGATTCACTTGAA
GAGTGAAAATGGTCAATTAGGTATTCTTATCGGAATCCAGCATTGGGGAAAGCCCTATAAAACAGAGTCCAATCAAACA
AAGTCCACTAAAGACAAGCCCACTCAGGACCCCATTGCCAGCCACTTCAAGGGCAAAGCTAAGACCCACACGGACTAGA
GCTAGTAAGGCTTTGCTACAAACCCAAAAACCTGAGCTCACCACACCAAAGAAGGTAAGGGTTATAAGATTCACTTGAA
GAGTGAAAATGGTCCAATTGAGGTATTCTTATCGGAATCCAGCATTGGGGAAAGCCCTATAAAACAGAGTCCAATCAAA
CAAAGTCCACTAAAGACAAGCCCACTCAGGACCCCATTGCCAGCCACTTCAAGGGCAAAGCTAAGACCCACACGGACTA
GAGCTAGTAAGGCTTTGCTACAAACCCAAAAACCTGAGCTCACCACACCAAAGAAGGAGTACTTTTATCATTTTAAGTA
GTGGGTCAGTATCCAGATTGATTCCTTATTGTAAAATCTTGTCATCCATGACCAGTGGATTAAAATTCATCTATGTTTC
ATAGATAATGGAAAAAATATTAGAGGACAATAGATGACTATGAAAAAAAGTTATTTATTAATATCATTTATTAATAATG
CATTTTAAGCAGATTTCCTGTTGTAATTTTTTTTTAGAAAATGTATTCTTTCCGAGGATATACATTCAAGTATTGAAAT
TTGTATTTTCTTTCCAGGAGGCCTTTATCCCCTAGCCTGGTTAACATAAAGACTGAGCTACCAGACCCAGATGAAGGTG
ATGATCCTCTAGGACCCTCACCAAGCTTAGATTTAGATGATGACAGTATCCGTAGTGCCCTTATATTGGGTTCTGATGA
TTTGGGACCTGTTGGTGGTAAGCTTCAGCTTCAGATGGAAGACCAGAGTGAAGGTAATGAATTTCTTGGTACAGAAAAT
AGTTTTGAATTGTAATTAAGAATATTTTCAGTAAAGAAAGTACCTTAGGTCACAGAAAGAAAGTCCTTTGGTGCATAAT
GGATATTTGTACAAGATGAAATTACATGGTGGCTGCAGGAACTTCCACTGTGGTATGCCCACAGAGGCACATTAAAACC
GTATCACAGTGTTAGTTACCTCCAGGCTCTATGTTTAATGCAACAGAATATCTACAGATGCCAGGAAACAGGTAAAAAG
TTCAATTCTCTTCATAAGAAAGTAAAGAAATAGGGGAATCTCTACAGCTTCTTTCATTGGAGAGTTCCTAAAGAAAGAT
TTTTAGTGTGAATGTTATCAAATGTCATTTTCATTCCAGGTTAAAATTAAGTGTAAAAAAATACACATGATCTTGCAGG
TGTGGTATTATAAATTTTGGTAAGAGAATTGAAGATGAAAAAATGACACTTTCTGGAAGAATAATAAATTACAAGAACA
GTTAGTGCTAGAATGTTTCTCATTCATAAGTCCTTTACATCAGTGCTTGACCAAGGAATTGTAGTGTTATTTCATCTTG
TGAAGTATATGGAGCTGGAGTAATGACACAGGGTTGTAGGTTGTGTTGGATCTCCTGGTCCTGTGTCTGGTCAAGTTAC
CATAGCACCAGTGGATTCAGCATGGTCTGGAACCCTTCTCTCGACGCCAGAACACAAGTGCATGAAGGGTTTGAATTTG
GCTATGAATGCAATTGCAATTGCTTAAATGATGTGGGTTAAGTTTGTGCAATTATTATGTTGAGGAAAGGTGAGGAATT
AAATCCTACTTTTCTGAGATAAGCAGTACTTTTATCATTTTAAGTAGTGGGTCAGTATCCAGATTGATTCCTTATTGTA
AAATCTTGTCATCCGAACCTGAGCTCACCACACCAAAGAAGGTAAGCAGTACTTTATCATTTTAAGTAGTGGGTCAGTA
TCCAGATTGATTCCTTATTGTAAAATCTTGTCATCCGAGTATCCAGATTGATTCCTTATTGTAAAATCTTGTCATCCAT
GACCAGTGGATTAAAATTCATCTATGTTTCATAGATAATGGAAAAAATATTAGAGGACAATAGATGACTATGAAAAAAA
GTTATTTATTAATATCATTTATTAATAATGCATTTTAAGCAGATTTCCTGTTGTAATTTTTTTTTAGAAAATGTATTCT
TTCCGAGGATATACATTCAAGTATTGAAATTTGTATTTTCTTTCCAGGAGCCTTTACCCCTGCCTGGTTAACATAAAGA

CTGAGCTACCAGACCCAGATGAAGGTGATGATCCTCTAGGACCCTCACCAAGCTTAGATTTAGATGATGACAGTATCCA
GTGCCCTTATATTGGGTTCTGATGATTTGGGACCTGTTGGTGGTAAGCTTCAGCTTCAGATGGAAGACCAGAGTGAAGG
TAATGAATTTCTTGGTACAGAAAATAGTTTTGAATTGTAATTAAGAATATTTTCAGTAAAGAAAGTACCTTAGGTCACA
GAAAGAAAGTCCTTTGGTGCATAATGGATATTTGTACAAGATGAAATTACATGGTGGCTGCAGGAACTTCCACTGTGGT
ATGCCCACAGAGGCACATTAAAACCGTATCACAGTGTTAGTTACCTCCAGGCTCTATGTTTAATGCAACAGAATATCTA
CAGATGCCAGGAAACAGGTAAAAAGTTCAATTCTCTTCATAAGAAAGTAAAGAAATAGGGGAATCTCTACAGCTTCTTT
CATTGGAGAGTTCCTAAAGAAAGATTTTTAGTGTGAATGTTATCAAATGTCATTTTCATTCCAGGTTAAAATTAAGTGT
AAAAAAATACACATGATCTTGCAGGTGTGGTATTATAAATTTTGGTAAGAGAATTGAAGATGAAAAAATGACACTTTCT
GGAAGAATAATAAATTACAAGAACAGTTAGTGCTAGAATGTTTCTCATTCATAAGTCCTTTACATCAGTGCTTGACCAA
GGAATTGTAGTGTTATTTCATCTTGTGAAGTATATGGAGCTGGAGTAATGACACAGGGTTGTAGGTTGTGTTGGATCTC
CTGGTCCTGTGTCTGGTCAAGTTACCATAGCACCAGTGGATTCAGCATGGTCTGGAACCCTTCTCTCGACGCCAGAACA
CAAGTGCATGAAGGGTTTGAATTTGGCTATGAATGCAATTGCAATTGCTTAAATGATGTGGGTTAAGTTTGTGCAATTA
TTATGTTGAGGAAAGGTGAGGAATTAAATCCTACTTTTCTGAGAAGGGGGGTGGTTTGTACTGGTTAAATATGTGGATT
AGATGCATAAAAAAAGAGAAATGGTAATGAAATGGGAAAAAACAGTTTTTGCAGGTGCGTTGTAGGGAAGGCAAATACT
AAGACAGTATTTGCTATTGGGCCAGGACAGGAGAAAGTGTGGGAGAGATCAAAAGTTCATGAGTGATAGGAGGGAAATG
TATGGCAGCTGGACAGACTGGAAGGAAATGCTGTTTTTTTTTGTGTGTGTGTGTTTCTAGTGATTAAGAAATAAACTGC
TTTATGTATTCTTCTTTAGTATGCTAATCTCAAACACTAATTTTGTACTGTCTTTTCCCTTTCAGGTGGATCTGAAGAT
TTAATGCTTGGCTACTCTTCTGGGTCTTCACCACCACCATTCCTTGCCCTAGAGCCACCTATTTCAGATACAGACTACA
CCTTTTCTCTTGATCACACGGAGGGCCTAAGTGATCTCTTTGATTTTAATTTCTGACCGCGCGGGGTTTAGTTCAGGGA
GTGTTTTGTAAAGGCTTTTGAGATCTTTTCATGCAAAATCAGTTAGATATAATCTGCATTAGCATTATTATTCTAATTA
TTGTTATTTTTAATATATATATTTTTTCTTATCACAATAATTCCTACAGACTTGCACCTCCATGACGAGAGTGATTTGA
ACCATAATTTCCTCTTCATGTATTTTATAGACCAAAGCTTGGTTTTTGTCTTGCTTAGAATCCCTCTATTTTTCTCTCC
TGTCAATATGGGTTCTATAGTCTATTATAGAAAGAAAAATAATCCAGGAATGAAATTATTTATATATAGATAAGAATTA
AGCACTGGTATTTGTTATCAGTGCAGAATATTTATATAAAGGAAATGTCATCGAAGGGACTTATCTGTGGTGACATTTC
TGACAGTTTAACTTGATACAGCATCTAGCTTTTGATGTAGCCATCCCCTCTCCATTCTTGTTTAAAGACAATTTTTATT
ATTTATTTTATTTTTTTCTTTGTAGAAGTTTTGAAATGAATGAGGAGGTGATAACAGACCCTAGAAATGGCACAGTTTT
TGAGTGGTCCAATCCAGTTCCACACGTGATTCTGCCTTCGGTGGCAATCTAGTTATTGCTTTCATTTCTATGAATGCTG
AGAGCATACTGTTTAATCATGCAGTTCATTTTGCATTCCTTGTATAGAGAACACTTCCCAAACTGCTGTACTATTGATA
TTTTTTAATATGATTGTAGTCATATTTTCCTATGCACTGTCTGTGGATATCATGGGGTATAAGACCCACAATTTTGTGC
TTAGATGCTGAGTGCTGCCCCTCATCCTTCCATTATCTTATGGATACACAAGGAAATTATTCTTGCTTTACTCTGAATG
ATTTATTTTTGTGCCAAGGAAGTGGCTGTCGGCATGACAGTAAATTTTCACTTGCTTTAAATAGTTCGTCCACAAATCA
CAAAAGCTGACACATTATCTCCAGTCAAGATGCCTTAATCAATATTTTAATTCTATTACTTGTATGGTATTTCTACTAT
GTCTTCACTGGTAAAAACAGTCATTCTATTCTGCCATTAAAGATAATTGAATATTTACAGTAGCAGATGGAGATTTTTT
GTATAATTGGGTATGGCAAAGAATTTTGTTCAAGTGTTAGAATTTACAGTGCCTGGGATGTGTGGAGGAGCAGCCAAGG
AAAACTAGGATTTTGTAATTTCATCCACACTAGTTGTGTAACAACTTCAAAGACATGGGATTAAATTGTCCAGTCCCAA
AGGCATTGTATTGAACATTTTGCTTTTCTGCTAAGGCTTGTAAAGCTTCTTTTTCTTCCACCTGACCCTCTCTTTTTGT
TTTCTTCTTTTGCATGTGTTAATTGTACATGATTTTGCTCCCAATTTCACTGCCATGCTGATACAGTGACATCAAAGTC
ACTTAGATGCAGAGCTCACTCAGATTTGTGCCAGACCCAGTGGACCTGAATTTCAACGCTCCAATATGGCAGTGCCAGT
AAATTTTATTTTTTTAATCAAGTGTAAAAGTTTATTGCTAACTGGTCTCAATCTCAAACTTGGATTTGTCCTTTGTATA
TTTATAGATTACAATTATTTTTATTATTATTTTTTTTTTATTTTTTTTTTTTATTTTTTTCATTTTTTGTTTGTTTTTGC
TTTTTAATTTTACAGGAAAATATTTTTAGCTTCTTTAATGACATTAACGATCGGTGATTAGCCATATAGTGTGTTGTGT
GTTATGTTTAAAGTGGGGGAAAAAAGATCACTTGCATACAAATCTGCATTGTACATGATAGGAAATGACTGTCAAGGTA
GTGCAGTGCTTAAAGACAAGTGGAGAAAACCAGAAGCCAATGGGATTTTATTCCATTTCCTGAGTTGAAGATGAGAGAT
TTTCCAATCCTCTCAGTTGATCATAGAAGAGAGAAGCTGCAAGTCTGGAAGGTGTGTCGTCATGAGTATGTGTGTGTGT

TTGTGTGTGTGCATGTGTGCATTTATGTATATATCTAGATGCAGGAAGTTGTCCCAAATGGCATGCAATCACTGATCAT
GTTACCTGAATATTGGCGCTCCAATTTTGCTACAAGTTGTATTGCTCATGTCTGAGGGAGACATCACAAAGATGTTTCT
AAAGTTTTTCATTTCATGTAAATTGATGGTTTTAAGTACAAATTATATTCATTCACTGACACGGCTGTGACAAGAAAAA
CCCAATGGTAGTTTGTTTTTATTCAAAATTGTTACTTTATTTTCAGTTTTGTACTGATGTTAGTGGTTGTCAGACAAAA
ATTCCCATACCAGTACTTTTCACAGCAGTGTCTGAATGACACATATTTGTTATAAAGATGGAAATAAGAGATATATGGA
ATAGATGATGTGATTTTGTTACCCTTTTACACATATAATGATCTTGGATATGAAAAGAAATTTGCATTAAAAACACTCT
CTCTCTCTCTCTCTCTTTCTCTCTCTCTCTCTCTCTCTCTCTCTCTTTCTCTCTCTCTCTCTCTCTCTCTTTCTCTT
TCTCTCTCTCNTGTGTGTGTGTGTGTGTGTGTGTGTGTGTGCGCACACACGCACAAGCATATACGCACAAAGAGTGGCCCA
AAAAAACATTACAGCAAATGTAAAAAAACGACTACCAAATGTACGAAAACGTGCACTAAAAAGTACCAAGGGATTCCAG
CCTGTCTCGAACACGTCGAATGGCACCGAAATTGAACAGCACATCTTCCGAGCGACGTTCGCGTATGTATGTTCTCGCC
CTCGCTCTGGCAATTTGTTCTTTCATAACCCTGATGCTGTAACAATGGCAGACTGGCGAGACGGGGCTGCTGAAGGAAC
ACCGTAAGACGCCGGCGAGATTGTCTTAAGTTTCATTCCGGAATCGCGTACGTCTGGCGAGATTGCGGGGTTGAGCGCG
CGCCGTTTTGGGCCTTGGTGCAGCATATTTGGTTTTGTGGGTGTTTGCGTTTGTTTGTGGGTGTGGGAGTATGTGCTTG
TTTGTGTGGGAGTATGTGTATGTGTGTGTTTGTCGGTGTGAGAGTATGTGTTTGCGGGAGTGGGAGTATGTGTTGTGGG
TGTGGGAGTATGTATATATGCTTGTTTGTGGATGTGGGAATATGTGTTTGTGGGTGTCGAAGTTTGTGTTTATGTGTGT
GGGAATATGTGTTTGTGTGTACAGGATAGGGAAATGGTTATGTTTGTGTGTTGTGCGTGTGCAGGTGATTTGTGTATAT
GTTTGTGTGTGTAAGTGTGTTCTTGCATGCGTTTGTGTATTGTTGTGTTGATGTGCGTGTGTTTATGTGTGTTCGAGGG
TTTGTATGTGTGCGTATGATAGATGTGTCTATATGATATATTTAAGTAATACAAAATGAATTTATAAAGTAATACATAT
AGAAAATGCTTATATACATACACAGTTGTGGTAGCACAATGCTTTAATGTAAATTCAAATATCAGTCGCTCTTTTTCGT
TTTCTCTCTCTCTCTCTCTCTCTCTCTCTCTCTCTCTCTCTCTCTCTCTCTCTCTCTCTCTCTCTCTCTCTCTCTCTCT
CTCTCTCTCTCTTCTTTCCTCTTCCTCCTCCTCCTCCTTTTCTTCTTCCTCCTCCTCCCGTTCTTCCTCCTGTTCC
TCTTCCCTTTCCTCCTCTTTCTCCTTTTCTTCCTCCTCCTCCTCCCTTTTTTCCTGCCTCTTCTTCCTCTTCTTCCCTT
TCTTCCTCTTCCTCCTTCGCCCGATCCCGAAGCAGAAGTCGCCGTTGCAGCTACTGAACCGACAGTGAATGTCAAACGC
TCTCAGCCGGCACATTAATATTTAGTGGTACCATTCTATCCTGCTCTCGCTCTCCTTCCTCCTCTCTCTCTCTCTCTC
TCTCTCTCTCTCTCTCTCTCTCTCTCTCTCTCTCTCTCTCTCTCTCTCTCTTTCTCTCTCTCTCTCTCTCTCTCTCTCT
CTCTCTCTCTCTCTCTCTCTCTCTCTGGCTCTCTCTCTCTCTCTCTCTCTCTCTCTTTCTCTCCCTCTTCATTTCTGTTTCCT
GATTCTTGTTTGTTTCCAGTTTATTTTTTGTATTCATTCTTTTCTGTTGCATTTTGGTAATTTTTCATGGTTTGTTTTT
TATAACCTTTTCTCTCGTAGTGTCTAGTATTCTTGTCATTATTTCTTTCTCTCTCTCTCCATGTGTCAGTTACTCTATT
TTTCGTTTCTCCTTTTCGTTTTGGCACATTTCTTATTTTTTCATGTTATATTCTTATTTGACTGTTTTGCATATCTTTG
CTTCATTTCATTCAACTCCTGATTCTTTTTCCACTCCATCTCCCTTCCTCTCTCTCTCTCTCTCTCTCTCTCTCTCTCT
CTCTCTCTCTCTCTCTCTCTCTCTCTCTCTCTCTCTCTCTTTCTCCCTTTTTCTCTCTCTCCTTCTTTAATATCCTTTTTC
TTCTTCTCCTGTATTTTCTTCTCCTCCATTCTCTTTCAGATAAATTCTCTCTCTCTCTCTCTCTCTTTCTCTCTCTCTC
TCTCTCTCTCTCTCTCTCTTTCTCTCTCTCTCTCTCTCTCTCTCTCTCTCTTTCTCTCTCTCTCTCTCTCTCTCTCTCT
CTCTTTCTCTCTCTTACTATCTGTTTATCTCTCCCTATTTATTATAACTATACATTTGCTTGTCTTCCATTTTATGTAT
GTTATTCTTTTTCGTATTTTTAGTTTGCTGAAATATATCTCTACCTTCTTATCCGTATACTTAAATAGTTAATTAACTA
TCATGGAAAGAGAGAAAATGTGGAAACCGTAAATATCTATGTCCCTCCCCTACTCGTGTATTTCTCTATATTCATTTTC
TTTTCTAATTTTCATACTGTTTTATATTTATCCATTCCTTCTCCCTTTCCCATTATGTGCAAACGGCCCTTTTAGTTTA
GCCAATCAGAGAGTTGGAGACATTAACCATGTCGTTTGAAACAAACTATATGAGTAAAGGAAATCTTCTCTCGTATCCA
GGGCAACCAATGAGAGCGAGGAATGACATCTCGGCTAGATCAAGGCCACCAATGAGAGCGAAGAAAGATCTCTCGGTTA
AATCAAAAGCGCCAATGGGAGCGAAGAATGACCTCCAGGTTAAATCAAGGTAACCAATAGGAGCGATAGAGAATAACGG
ACTATTGTTTCAGCTGCAGGTATTTCGAAACAGCTAAAAAAAATAATTTAGGAGAATTAACATAGAGATGACAGTCACC
GCCCAATCTCTTTGATGAAAATCCAATCAGTCGACAGAATGGCCCAATCTCTTTGATGAAAATCCAATCAGTCGACAGA
ATCCATCAGATTTTTGTTTGACTATTCAGCGCCTTTTTAAAAACGCGAAAGTCAACTCTATTCATTAATGTTCTCAAAA
GAGAGAGAGAGAAAAAAAACTTTTTCTGCAAAAACAAATAAAAATGCTTGAAAATTCAGGTAAATACGATCAAAACTTA

CTGCAATAAATATTTTTTCTAACACCGTTTAAATTTTTTGAATGACAATGAAACGAAAAGTTACATTGTGTATATACAT
ACAT
